# ATP-dependent one-dimensional movement maintains immune homeostasis by suppressing spontaneous MDA5 filament assembly

**DOI:** 10.1038/s41422-025-01183-8

**Published:** 2025-09-19

**Authors:** Xiao-Peng Han, Ming Rao, Yu Chang, Jun-Yan Zhu, Jun Cheng, Yu-Ting Li, Wu Qiong, Si-Chao Ye, Qiurong Zhang, Shao-Qing Zhang, Ling-Ling Chen, Fajian Hou, Jin Zhong, Jiaquan Liu

**Affiliations:** 1 State Key Laboratory of RNA Innovation, Science and Engineering, Shanghai Key Laboratory of Molecular Andrology, CAS Center for Excellence in Molecular Cell Science, Shanghai Institute of Biochemistry and Cell Biology, University of Chinese Academy of Sciences, Chinese Academy of Sciences, Shanghai, China; 2 Shanghai Institute of Immunity and Infection, Chinese Academy of Sciences, Shanghai, China; 3 School of Life Science and Technology, ShanghaiTech University, Shanghai, China; 4 Infectious Disease Research Center, Shanghai Institute of Materia Medica, Chinese Academy of Sciences, Shanghai, China; 5 New Cornerstone Science Laboratory, Shenzhen, Guangdong, China; 6 Key Laboratory of Systems Health Science of Zhejiang Province, School of Life Science, Hangzhou Institute for Advanced Study, University of Chinese Academy of Sciences, Hangzhou, Zhejiang China

**Keywords:** RIG-I-like receptors, Single-molecule biophysics

## Abstract

MDA5 is a RIG-I-like receptor (RLR) that recognizes viral double-stranded RNA (dsRNA) to initiate the innate immune response. Its activation requires filament formation along the dsRNA, which triggers the oligomerization of N-terminal caspase activation and recruitment domains. The ATPase activity of MDA5 is critical for immune homeostasis, likely by regulating filament assembly. However, the molecular basis underlying this process remains poorly understood. Here, we show that MDA5 operates as an ATP-hydrolysis-driven motor that translocates along dsRNA in a one-dimensional (1D) manner. Multiple MDA5 motors can cooperatively load onto a single dsRNA, but their movements rarely synchronize, inhibiting spontaneous filament formation and activation. LGP2, a key regulator of MDA5 signaling, recognizes MDA5 motors and blocks their movement, thereby promoting filament assembly through a translocation-directed mechanism. This unique assembly strategy underscores the role of 1D motion in higher-order protein oligomerization and reveals a novel mechanism for maintaining immune homeostasis.

## Introduction

The innate immune system is the first line of host defense against microbial pathogens and relies on pattern-recognition receptors (PRRs) that identify pathogen-associated molecular patterns (PAMPs).^1,2^ Among these PRRs, RIG-I-like receptors (RLRs) are cytosolic viral RNA sensors found in mammals, including RIG-I, MDA5, and LGP2. All three of these human RLRs share a highly similar structure, containing a DExD/H-box helicase domain and a CTD that both are crucial for RNA recognition.^1–3^ RIG-I and MDA5 have two N-terminal caspase activation and recruitment domains (CARDs) that can mediate downstream signal transduction, with intrinsically disordered regions connecting the CARDs to the helicase domains. LGP2 lacks the CARDs and is generally believed to regulate the functions of RIG-I and MDA5.^4^

The antiviral signaling begins with viral RNA recognition by the RLR sensors. Short dsRNA with a triphosphate group at the 5’ blunt end (5’-ppp) serves as an agonist for RIG-I, while MDA5 prefers long dsRNA fragments (>1 kb) such as picornavirus replicative-form dsRNA.^5^ Signaling downstream of RLRs requires the adaptor protein mitochondrial antiviral signaling protein (MAVS, also known as IPS-1, CARDIF, and VISA),^6–9^ which is localized to the mitochondrial outer membrane and comprises a CARD domain at its N terminus. When RIG-I or MDA5 is activated by viral RNAs, CARDs from proximate RLR proteins assemble into tetramers that directly interact with MAVS CARD, leading to the formation of MAVS filament.^10,11^ The MAVS filament further recruits TRAF molecules and activates the downstream signaling pathway.^12^

The proximity-induced higher-order assemblies have been regarded as a fundamental principle governing immune signaling.^13^ To maintain cellular immune homeostasis, it is crucial to suppress the spontaneous assembly of CARDs within RLRs.^1,2^ Without viral RNA, RIG-I remains in an auto-repressed state, with its tandem CARDs bound to the helicase domain.^14,15^ This configuration serves as a physical barrier to prevent CARDs from forming tetramers, thus blocking signaling to MAVS.^16,17^ Binding to viral dsRNA with a 5’-ppp induces a conformational rearrangement within RIG-I and releases the CARDs.^14,15^ By contrast, MDA5 lacks intrinsic CARD–helicase interactions^11,17^ and relies on its ATP hydrolysis activity to maintain immune homeostasis. Therefore, mutations that disrupt ATPase of MDA5 are associated with autoimmune diseases.^18–20^ Early studies have shown that the helicase and CTD domains of MDA5 form a ring-like clamp around dsRNA,^21^ with multiple MDA5 rings arranged head-to-tail along the dsRNA axis, potentially creating a filamentous, highly organized structure.^22–25^ It has been further demonstrated that the assembly of an MDA5 filament leads to the oligomerization of CARDs in close proximity, thereby activating MAVS.^26,27^ Although the ATP cycle of MDA5 is proposed to regulate filament formation, the mechanisms underlying this control remain elusive.^18,26,28^

In recent decades, significant observations have emerged regarding proteins moving along the nucleic acid backbone in a one-dimensional (1D) manner. These movements involve various mechanisms such as ATP-hydrolysis-driven translocation, passive sliding/diffusion, intersegmental transfer, or hopping/jumping.^29,30^ Additional evidence indicates extensive utilization of 1D motions by DNA-binding proteins during DNA repair, DNA replication, and genome maintenance. Their biological functions are often distance-related, for example, facilitating target search,^31,32^ enhancing processivity,^33,34^ enabling long-range communications,^35^ and driving loop extrusion.^36–38^ Several dsRNA-binding proteins involved in the innate immune response, including RIG-I, LGP2, transactivation response RNA binding protein (TRBP), and protein activator of protein kinase R (PACT), have also been reported to perform 1D movement along dsRNA.^39–41^ However, their potential impact on immune homeostasis remains largely unknown.

In this study, we employed single-molecule imaging to monitor MDA5 oligomer assembly in real time. Our results reveal that MDA5 functions as an ATP-dependent dsRNA motor, where the movement of a single molecule drives the cooperative loading of multiple proteins traveling in the same direction. These motors hydrolyze ATP asynchronously and translocate independently. LGP2 binds to mobile MDA5 and arrests their motions, thereby promoting filament formation in a translocation-directed, dsRNA length-dependent manner. When this 1D motion is absent, MDA5 becomes static, filaments assemble spontaneously on short duplexes, and aberrant activation occurs. These findings provide new insights into how dsRNA sensors control higher-order assemblies through 1D motion.

## Results

### MDA5 is a dsRNA motor powered by ATP

**Fig. 1:**
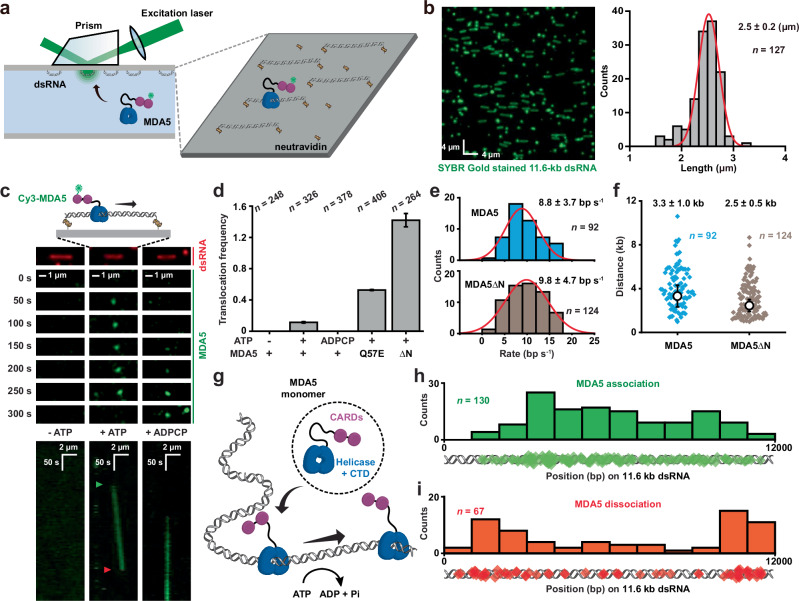
MDA5 is an ATP-hydrolysis-driven motor on dsRNA. **a** A schematic illustration of dsRNA and MDA5 observations by prism-based smTIRF microscopy. **b** Left: representative 11.6-kb dsRNA visualized by smTIRF microscopy in the absence of flow. The dsRNA was stained with SYBR Gold and a 42.7 × 42.7 µm field of view is shown. Right: the length distribution of the 11.6-kb dsRNA observed by smTIRF microscopy (*n* = number of dsRNA molecules). The data were fit with a Gaussian distribution that determined the mean ± SD. The contour length of an 11.6-kb dsRNA is calculated to be 3.3 µm. **c** Top: representative fluorescent images and a schematic illustration showing the translocation of a Cy3-MDA5 (3 nM) motor on 11.6-kb dsRNA with ATP (middle, +ATP), and the absence of any MDA5 motors without ATP or with ADPCP (left and right, –ATP and +ADPCP). SYBR Gold-stained 11.6-kb dsRNA is shown in red and Cy3-MDA5 at various times is shown in green. Bottom: representative kymographs of Cy3-MDA5 on dsRNA under various conditions. Kymographs were generated by image stacking (see Supplementary information, Fig. S2a). Green and orange arrowheads indicate the association and dissociation of MDA5 motor on dsRNA, respectively. **d** The frequency of MDA5, MDA5(Q57E) or MDA5ΔN motor (3 nM) translocation on 11.6-kb dsRNA under various conditions observed by smTIRF (mean ± SD; *n* = number of dsRNA molecules). **e** Histogram of binned MDA5 (top) and MDA5ΔN (bottom) translocation rates that were fit to Gaussian function to derive the average rates (mean ± SD; *n* = number of events). **f** Distribution of MDA5 and MDA5ΔN translocation distance. Diamonds represent individual events and open circles represent the mean processivity derived by single exponential decay fitting (mean ± SE; *n* = number of events). **g** A schematic illustration showing the translocation of a single MDA5 molecule along dsRNA backbone. **h** Distribution of the starting positions for MDA5 translocation on dsRNA (*n* = number of events). Diamonds represent individual starting events. **i** Distribution of the ending positions for MDA5 translocation on dsRNA (*n* = number of events). Diamonds represent individual ending events. All single-molecule studies were performed at least two separate times.

We exploited prism-based single-molecule total internal reflection fluorescence (smTIRF) microscopy^42,43^ to visualize individual MDA5 molecules on dsRNA in real-time. Single 11.6-kb dsRNA molecules were constructed by in vitro transcription, stretched across a passivated custom-made flow cell surface by laminar flow and linked at both ends via biotin-neutravidin (Fig. 1a, b; Supplementary information, Fig. S1a, b and Table S1). Human MDA5 protein was purified and labeled with a Cy3 fluorophore similar to our previous studies^43^ (Supplementary information, Fig. S1c, d and Table S2). Injection of Cy3-MDA5 with ATP into the flow cell resulted in numerous single particles exhibiting 1D movement along the dsRNA (Fig. 1c, +ATP; Supplementary information, Fig. S2a, Video S1 and Table S3). Interestingly, nearly all of MDA5 molecules moved unidirectionally without a reversal of direction, resembling the motions of a mechanochemical motor or an enzyme translocating along a double-stranded DNA (dsDNA) molecule^32,44,45^ (Fig. 1c; Supplementary information, Fig. S2a). The translocation of MDA5 was completely abolished in conditions lacking ATP or when ATP was substituted by a non-hydrolysable ATP-analog adenosyl-methylene-triphosphate (ADPCP) (Fig. 1c, d, –ATP and +ADPCP), demonstrating that ATP hydrolysis is required during the 1D movement along dsRNA. In alignment with the single-molecule analysis, ATPase activity of MDA5 was stimulated upon the addition of 11.6-kb dsRNA (k_cat_ = 1.1 ± 0.1 s^–1^), but not ssRNA (Supplementary information, Fig. S2b, c). These observations directly show that an individual MDA5 molecule operates as a motor, translocating along dsRNA using the energy derived from ATP hydrolysis.

While the helicase domain and CTD of MDA5 are responsible for substrate recognition,^21^ it remains unclear whether the CARDs participate in the 1D movement. A mutant MDA5(Q57E), which had impaired CARDs–CARDs interactions,^11^ and CARDs-deleted MDA5 (MDA5ΔN) proteins were purified, labeled and introduced into smTIRF (Supplementary information, Fig. S1c, d). Both the mutant and truncated proteins largely retained the dsRNA-dependent ATPase activities (Supplementary information, Fig. S2c). Remarkably, the frequencies of MDA5(Q57E) and MDA5ΔN translocation events were ~5-fold and 12-fold higher, respectively, compared to wild-type MDA5 (Fig. 1d; Supplementary information, Table S3), whereas the translocation rates and processivities between MDA5 and MDA5ΔN appeared nearly identical (Fig. 1e, f; Supplementary information, Fig. S2d). As a control, immobile MDA5 exhibited a near-zero translocation rate (Supplementary information, Fig. S2e, f). These findings suggest that MDA5 motor always initiates translocation as a monomer, and it is likely that the CARDs–CARDs interactions occur between free MDA5 molecules, leading to a decrease in monomeric protein concentration for wild-type MDA5 (Fig. 1g).

The initial binding of the MDA5 motor to its substrate appeared to occur randomly across the entire length of dsRNA (Fig. 1c, h), indicating that MDA5 recognizes internal dsRNA backbone without dependence on specific sequences. By contrast, most of the dissociation positions of MDA5 were at or very near the ends of dsRNA, suggesting that the motor is released upon encountering an RNA end (Fig. 1c, i). With the dsRNA ends attached to the surface by highly stable biotin-neutravidin interactions, these dissociation events likely resulted from the opening of the MDA5 ring-like structure. Taken together, these results are consistent with the notion that MDA5 motors mainly recognize internal regions within dsRNA substrates.

### MDA5 motors oligomerize without forming filamentous structures

**Fig. 2:**
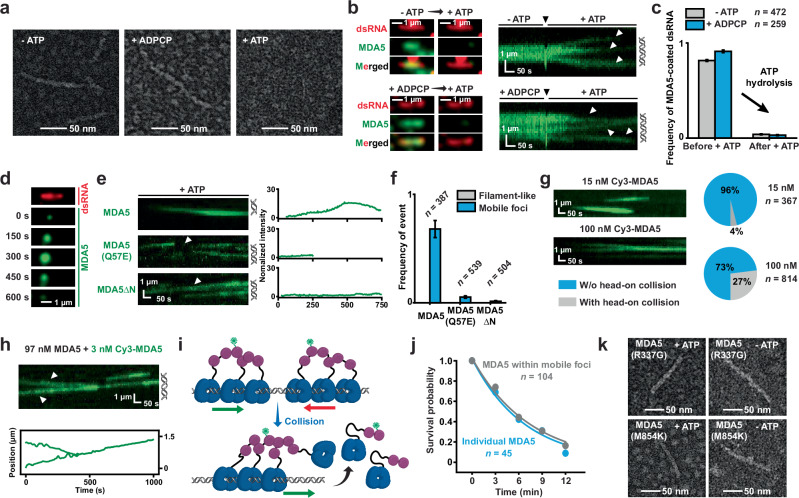
1D translocation promotes the formation of ATM clusters. **a** Representative EM images showing the formation of MDA5 filaments without ATP or with ADPCP, and the absence of MDA5 filaments with ATP. **b** Left: representative fluorescent images showing the dissociation of MDA5 through ATP hydrolysis. SYBR Gold stained 11.6-kb dsRNA is shown in red and Cy3-MDA5 (–ATP: 60 nM; +ADPCP: 15 nM) is shown in green. The arrows indicate buffer exchange. Right: representative kymographs showing the MDA5 dissociation through 1D translocation. Cy3-MDA5 is shown in green. Black arrowheads indicate the time of buffer exchange. White arrowheads indicate the MDA5 translocation events. Positions of dsRNA are shown adjacent to the right of kymographs. **c** Frequency of MDA5-coated dsRNA before and after buffer exchange (mean ± SD; *n* = number of dsRNA molecules). **d** Representative fluorescent images showing the cooperative binding of multiple Cy3-MDA5 motors and the formation of mobile foci. SYBR Gold-stained 11.6-kb dsRNA is shown in red and Cy3-MDA5 (15 nM) at various times is shown in green. **e** Representative kymographs (left) and time-dependent fluorescent intensities (right) showing the cooperative binding of Cy3-MDA5, Cy3-MDA5(Q57E) and Cy3-MDA5ΔN motors (15 nM) on a dsRNA substrate. Cy3-MDA5 is shown in green. Arrowheads indicate the MDA5 molecules used for fluorescent intensity plots. Positions of dsRNA are shown adjacent to the right of kymographs. **f** The frequency of filament-like structure and mobile foci formation by MDA5, MDA5(Q57E) and MDA5ΔN (mean ± SD; *n* = number of dsRNA molecules). **g** Left: representative kymographs showing the mobile MDA5 foci at various protein concentrations. Right: pie charts showing the distributions of head-on collision events of mobile MDA5 foci at various concentrations (*n* = total number of mobile MDA5 foci examined). **h** Representative kymograph (top) and single-particle trajectory (bottom) showing the behavior of individual MDA5 motors within colliding foci. Arrowheads indicate the MDA5 molecules used for trajectory plots. **i** A schematic illustration showing MDA5 motors displacing each other during a collision event. **j** Survival probability of individual MDA5 and MDA5 within ATM clusters (*n* = number of events examined). **k** Representative EM images showing the formation of MDA5(R337G) and MDA5(M854K) filaments with or without ATP. All single-molecule studies were performed at least two separate times.

Previous studies have shown that MDA5 proteins form filaments on dsRNA through helicase–helicase interactions.^22,26,28^ However, these higher-order structures become unstable under conditions that allow ATP hydrolysis,^22,23^ as also evident from electron microscopy (EM) images of negatively stained MDA5–dsRNA complexes (Fig. 2a). We employed a two-step imaging approach to investigate the effect of ATP hydrolysis on pre-bound MDA5 (Fig. 2b, c; Materials and Methods). Initially, a large number of 11.6-kb dsRNA molecules were coated with immobile Cy3-MDA5, either without ATP or with ADPCP (Fig. 2b, c). ATP hydrolysis was subsequently triggered by introducing ATP and washing out unbound proteins through buffer exchange (Fig. 2b, c). Notably, the MDA5 molecules rapidly dissociated upon ATP hydrolysis, accompanied by extensive translocation events (Fig. 2b, c). These results suggest that ATPase activity and the 1D motions of MDA5 are unlikely to be synchronized.

When Cy3-MDA5 was injected with ATP at a protein concentration that allowed multiple motors to load onto a single substrate, we observed MDA5 molecules forming foci while simultaneously translocating along the 11.6-kb dsRNA, with their assembly and disassembly being highly dynamic (Fig. 2d, e; Supplementary information, Fig. S3a, b and Video S2). The fluorescent intensities of an MDA5 focus often increased more than 10-fold compared to that of a single protein, suggesting these foci contained at least dozens of MDA5 molecules (Fig. 2e; Supplementary information, Fig. S3a). These foci formed internally along the 11.6-kb dsRNA during protein translocation, failed to appear without ATP hydrolysis, and rapidly dissolved upon reaching a dsRNA end (Fig. 2d, e; Supplementary information, Fig. S3c and Video S2). A fraction of MDA5 foci translocation events (34% of observed events; *n* = 91/269) resulted in the displacement of neutravidin from RNA ends, an ATP-dependent activity reported in a recent study,^46^ leading to the withdrawal of the stretched dsRNA (Supplementary information, Fig. 3b, bottom). Collectively, these observations suggest that mobile foci represent MDA5 oligomers that form through cooperative binding during 1D translocation.

**Fig. 3:**
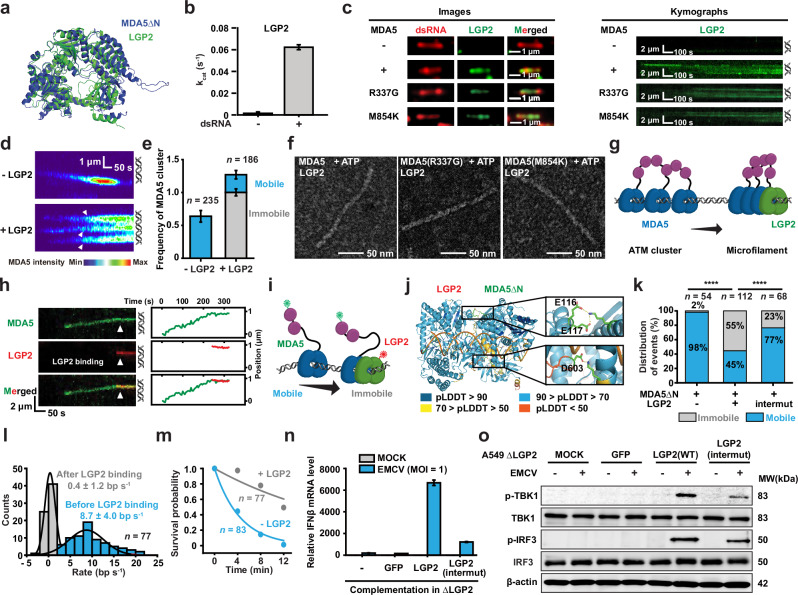
LGP2 inhibits MDA5 translocation to induce microfilament assembly. **a** AlphaFold predicted structural alignment of human MDA5ΔN and LGP2 proteins. **b** The turnover numbers (k_cat_) of LGP2 ATPase using 11.6-kb dsRNA substrate (error bars: mean ± SE). **c** Representative images (left) and kymographs (right) showing the binding of 60 nM Cy3-LGP2 on dsRNA under various conditions. SYBR Gold stained 11.6-kb dsRNA is shown in red and Cy3-LGP2 is shown in green. Positions of dsRNA are shown adjacent to the right of kymographs. **d** Representative kymographs showing the formation of Cy3-MDA5 ATM clusters (10 nM) in the absence or presence of 10 nM LGP2. Positions of dsRNA are shown adjacent to the right of kymographs. Fluorescent intensities in kymographs were normalized to generate the heatmaps. White arrowheads indicate the transition of mobile ATM cluster into immobile one. **e** The frequency of mobile (blue) and immobile (grey) MDA5 ATM cluster in the absence/presence of LGP2 (mean ± SD; *n* = number of dsRNA molecules). **f** Representative EM images showing the formation of MDA5–LGP2 filaments with ATP. **g** A schematic illustration showing the conversion from an ATM cluster to a microfilament induced by LGP2 binding. **h** Representative kymographs (left) and single-particle trajectories (right) showing a Cy5-LGP2 (3 nM) binding to a Cy3-MDA5 motor (3 nM). Cy3-MDA5 is shown in green and Cy5-LGP2 is shown in red. The merged kymograph is generated by overlaying both channels. Arrowheads indicate the association of LGP2 with MDA5. Positions of dsRNA are shown adjacent to the right of kymographs. **i** A schematic illustration of LGP2 binding resulting in a stop in MDA5 translocation. **j** AlphaFold model of an MDA5ΔN–LGP2 complex on a 42-bp dsRNA substrate. The closeup shows the key contacts between MDA5ΔN and LGP2. **k** Proportion distribution of MDA5ΔN translocation (mobile) and LGP2-induced stop (immobile) events under various conditions (*n* = total number of events). The results of two-sided pairwise comparisons using Fisher’s exact test are shown (****P < 0.0001). **l** Histogram of binned MDA5ΔN translocation rates before and after LGP2 binding. Data were fit to Gaussian function to derive the average rates (mean ± SD; *n* = number of events). **m** Survival probability of MDA5ΔN and MDA5ΔN–LGP2 molecules on dsRNA (*n* = number of events examined). **n** Relative IFNβ mRNA levels showing the MDA5 signaling activities in ΔLGP2 cells after 12 h EMCV infection. IFNβ productions were induced upon EMCV infections. MOI: multiplicity of infection. **o** Immunoblotting showing the EMCV-induced phosphorylation of TBK1, IRF3 in ΔLGP2 cells. p-TBK1: phosphorylated TBK1, p-IRF3: phosphorylated IRF3. All single-molecule studies were performed at least two separate times. All cell-based assays were repeated independently at three times with similar results. Error bars represent SEM between measurements, centered on the mean of a single experiment.

Wild-type MDA5 was then replaced with mutant or truncated proteins to probe the interactions essential for cooperative binding. Surprisingly, although many independent MDA5(Q57E) and MDA5ΔN translocation events were observed on a single dsRNA, they failed to form static, filament-like structures, and only a few mobile foci were detected (Fig. 2e, f). These data suggest that the CARDs–CARDs interactions are required for the oligomerization of mobile MDA5. To further characterize the nature of these mobile foci, we performed collision analysis using single-molecule tracking. At low protein concentration (15 nM), MDA5 motors tended to incorporate directly into existing foci, and head-on collision events were rarely observed (Fig. 2g; Supplementary information, Fig. S3d). By contrast, mobile foci frequently encountered each other at elevated protein concentration (100 nM) (Fig. 2g; Supplementary information, Fig. S3d). We tracked individual motors within colliding foci by mixing unlabeled and Cy3-labeled MDA5 (Fig. 2h; Supplementary information, Fig. S3e). Remarkably, MDA5 retained motor function despite at higher protein concentrations (100 nM, Fig. 2h; Supplementary information, Fig. S3e). Collisions between mobile MDA5 in opposite directions did not stop translocation or promote filament assembly; instead, the foci competed with each other, with one displacing the other and allowing unidirectional movement to persist (Fig. 2h, i; Supplementary information, Fig. S3e). The lifetimes of individual MDA5 within non-collision foci were identical to those of single motors (Fig. 2j). Together, these findings support the idea that mobile foci are composed of multiple independent MDA5 motors moving in the same direction, as stable filaments are unlikely to be dislodged. Given their distinct properties relative to static filaments, we refer to these structures as ATP-dependent mobile (ATM) clusters.

One might imagine that MDA5 motors within an ATM cluster move at unsynchronized speeds. This could displace adjacent molecules or create persistent gaps, thereby limiting protein–protein interactions at the filament-forming interface. By contrast, immobile MDA5 can establish stable helicase–helicase contacts, thereby allowing spontaneous filament assembly (Fig. 2a). To examine this hypothesis, we purified and fluorescently labeled two ATPase-deficient mutants, MDA5(R337G) and MDA5(M854K), both of which are associated with autoimmune diseases in humans^18–20^ (Supplementary information, Fig. S1c, d). Negative-stain EM images of MDA5(R337G)-dsRNA and MDA5(M854K)-dsRNA complexes confirmed their ATPase deficiency, as filaments formed regardless of ATP presence, consistent with previous findings (Fig. 2k).^18,20^ Unlike wild-type MDA5 in smTIRF experiments, both mutants statically coated the 11.6-kb dsRNA with filament-like structures in the presence of ATP, and neither MDA5(R337G) nor MDA5(M854K) was able to translocate along the dsRNA to form ATM clusters (Supplementary information, Fig. S3f–h). These results suggest that ATP-hydrolysis-driven 1D translocation modulates MDA5 oligomerization by suppressing spontaneous filament assembly and promoting the formation of ATM clusters.

### LGP2 induces microfilament formation by turning the MDA5 motor off

LGP2, normally present at low levels in cells but accumulating upon interferon stimulation, is essential for the MDA5-dependent immune response to both viral infections and endogenous RNAs.^4,47–50^ Previous studies suggest that LGP2’s structure is similar to that of an MDA5 motor lacking CARDs (Fig. 3a) and that LGP2 proteins are incorporated into MDA5 filaments, leading to the formation of numerous shorter filaments with greater agonistic activity.^41,46,48,51,52^ While the 1D movement of MDA5 inhibits spontaneous filament formation, it is unclear whether this motion also regulates the LGP2-dependent filament assembly. We purified recombinant LGP2 and found that it also maintained a dsRNA-dependent ATPase activity (Fig. 3b; Supplementary information, Fig. S1c, d), although the rate of ATP hydrolysis by LGP2 was ~20 times slower than that of MDA5 (k_cat_ = 0.062 ± 0.002 s^–1^). Cy3-LGP2 was observed to bind the 11.6-kb dsRNA in an MDA5-dependent manner, although MDA5’s ATPase activity was not required for this interplay (Fig. 3c). Notably, no processive LGP2 translocation events were detected (Fig. 3c).

Addition of LGP2 increased the frequency of MDA5 cluster on 11.6-kb dsRNA by ~2-fold, with most of these oligomers becoming immobile and more stable than ATM clusters (Fig. 3d, e; compare –LGP2 with +LGP2). Notably, the immobile oligomers formed after a brief 1D movement along dsRNA and an increase in fluorescent intensity (Fig. 3d, e), suggesting that they likely originated from ATM clusters on dsRNA. We envision that these LGP2-dependent immobile MDA5 oligomers correspond to the MDA5–LGP2 microfilaments observed in previous studies, but they cannot be resolved due to the limited spatial resolution.^51^ Indeed, EM imaging of negatively stained MDA5–dsRNA complexes in the presence of LGP2 revealed microfilament formation under conditions that allow ATP hydrolysis (compare Fig. 2a with Fig. 3f), consistent with a recent study.^46^ These observations suggest that LGP2 can transform an MDA5 ATM cluster into a microfilament (Fig. 3g).

Two-colored single-molecule analysis of Cy3-MDA5 and Cy5-LGP2 revealed that LGP2 was recruited by MDA5, as LGP2 consistently appeared adjacent to wild-type or mutant MDA5 on the dsRNA (Fig. 3h; Supplementary information, Fig. S4a, b). Remarkably, LGP2 binding appeared to immediately stop the MDA5 translocation, leading to the formation of an immobile MDA5–LGP2 complex on the dsRNA (Fig. 3h, i; Supplementary information, Fig. S4a). Immobile complexes were still observed when wild-type MDA5 was substituted by MDA5ΔN, indicating that the interactions depend on helicase–helicase contacts (Supplementary information, Fig. S4c). Using AlphaFold3, we modeled the interaction interfaces and found that several residues in LGP2 (E116, E117 and D603) seemed to be important for the interactions (Fig. 3j). A mutant LGP2, LGP2(E116A, E117A, D603A), hereafter referred to as LGP2(intermut), was purified, shown to retain dsRNA-dependent ATPase activity (Supplementary information, Fig. S4d) and introduced into smTIRF. The translocation events of MDA5ΔN were found to be highly processive in the absence of LGP2, whereas the addition of LGP2 resulted in a stop in most of MDA5ΔN translocation events (55%, Fig. 3k). Substitution of wild-type LGP2 by LGP2(intermut) significantly reduced the frequency of immobile events (23%, Fig. 3k), suggesting that the three mutations at interface could diminish MDA5–LGP2 interactions. The MDA5ΔN–LGP2 interactions had completely shut the motor down and extended its lifetime on dsRNA (Fig. 3l, m), whereas the initial positions of MDA5ΔN–LGP2 complex formation were randomly across the length of a dsRNA (Supplementary information, Fig. S4e).

To further reveal the essential roles of the MDA5–LGP2 interfaces in cellular antiviral immune response, we generated MDA5 or LGP2 knockout A549 cells using CRISPR/Cas9 (referred to as ΔMDA5 and ΔLGP2, respectively, Supplementary information, Fig. S4f). Interferon mRNAs were then measured following encephalomyocarditis virus (EMCV) infections. Compared with wild-type A549, both ΔMDA5 and ΔLGP2 cells showed significant deficiencies in interferon production (Supplementary information, Fig. S4g), consistent with previous studies demonstrating that both RLRs are required for signaling.^4,48–51^ Complementation of wild-type LGP2 in ΔLGP2 cells enhanced the expression of interferon and interferon-stimulated genes (ISGs), accompanied by increased phosphorylation of TANK-binding kinase 1 (TBK1) and interferon regulatory factor 3 (IRF3) that function downstream of the MDA5-MAVS axis, indicating robust MDA5-dependent signaling (Fig. 3n, o; Supplementary information, Fig. S4h, i). By contrast, replacing LGP2 with LGP2(intermut) resulted in a marked reduction in interferon and ISGs expressions, as well as diminished phosphorylation of TBK1 and IRF3, underscoring the importance of the MDA5–LGP2 interfaces for signaling (Fig. 3n, o; Supplementary information, Fig. S4h, i). Overall, these results strongly suggest that LGP2 binds to an MDA5 motor to pause its 1D movement; this alteration in motion likely removes gaps between MDA5 motors and induces microfilament formation (Fig. 3g).

### 1D movement as a regulatory switch for MDA5 signaling

**Fig. 4:**
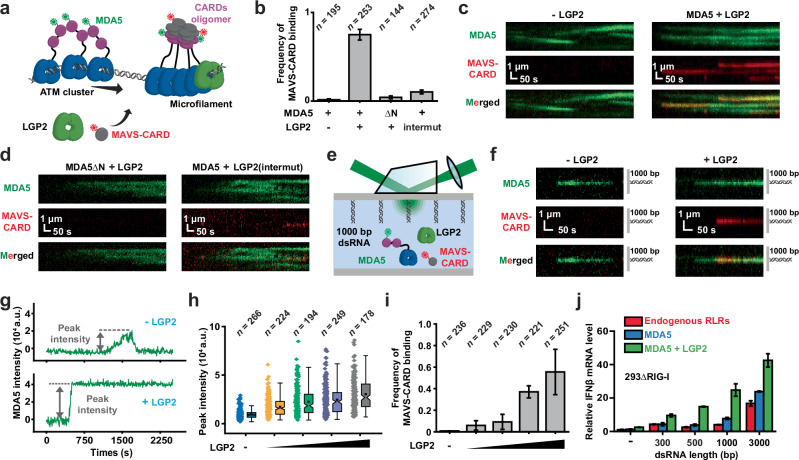
MDA5–LGP2 microfilaments, but not ATM clusters, mediate downstream signaling. **a** A schematic illustration of the MAVS-CARD recruitment experiment. **b** The frequency of MAVS-CARD recruitment by MDA5 and MDA5ΔN under various conditions (mean ± SD; *n* = number of dsRNA molecules). **c** Representative kymographs showing the recruitments of AF647-MAVS-CARD (100 nM) by Cy3-MDA5 (20 nM) in the absence or presence of LGP2 (20 nM). Cy3-MDA5 is shown in green and AF647-MAVS-CARD is shown in red. **d** Representative kymographs showing the recruitments of AF647-MAVS-CARD (100 nM) by Cy3-MDA5ΔN or Cy3-MDA5 (20 nM) in the presence of LGP2 or LGP2(intermut) (20 nM). Cy3-MDA5 is shown in green and AF647-MAVS-CARD is shown in red. **e** A schematic illustration showing the two-colored single-molecule co-localization assay. **f** Representative kymographs showing the formation of MDA5-LGP2 microfilaments on a 1000-bp dsRNA substrate using two-colored single-molecule co-localization assay. **g** Representative trajectories showing the formation of an ATM cluster without LGP2, and the formation of a microfilament with LGP2 on a 1000-bp dsRNA substrate. The dotted line indicates the peak fluorescence intensity. **h** The distribution of peak fluorescence intensity of MDA5 (15 nM) under various concentrations of LGP2 (0 nM, 3 nM, 5 nM, 10 nM, and 15 nM) on 1000-bp dsRNA substrate (*n* = MDA5 binding events). **i** The frequency of MAVS-CARD recruitment by MDA5 (15 nM) and various concentrations of LGP2 (0 nM, 3 nM, 5 nM, 10 nM, and 15 nM) on a 1000-bp dsRNA substrate (mean ± SD; *n* = number of dsRNA molecules). **j** Column plots of relative IFNβ mRNA levels showing the MDA5 signaling activity under various conditions. 293∆RIG-I (endogenous RLRs), 293∆RIG-I with MDA5 overexpression (MDA5), and 293∆RIG-I with MDA5 and LGP2 co-overexpression (MDA5+LGP2) were stimulated with dsRNA substrates of different lengths. All single molecule studies were performed at least two separate times. All cell-based assays were repeated independently at three times with similar results. Error bars represent SEM between measurements, centered on the mean of a single experiment.

The essential role of LGP2 in the antiviral immune response suggests that modulation of 1D movement may represent a unique regulatory mode for toggling MDA5 signaling activity. To test this hypothesis, we purified and labeled MAVS-CARD with Alexa Fluor 647 (AF647) (Supplementary information, Fig. S1c, d), and co-injected Cy3-MDA5, LGP2 with AF647-MAVS-CARD proteins to monitor their real-time interactions on 11.6-kb dsRNA (Fig. 4a). In the absence of LGP2, a number of ATM clusters were observed translocating along dsRNA, while no MAVS-CARD signal was detected (Fig. 4b, c; Supplementary information, Fig. S5a, b). Addition of LGP2 resulted in numerous MAVS-CARD molecules that were co-localized with immobile MDA5 (Fig. 4b, c; Supplementary information, Fig. S5a, b). Further validation revealed that the recruitment of MAVS-CARD required MDA5’s CARDs as well as the interactions between MDA5 and LGP2, as replacing MDA5 with MDA5ΔN, or substitution of LGP2 by LGP2(intermut) resulted in significantly decreased MAVS-CARD binding on dsRNA (Fig. 4b, d; Supplementary information, Fig. S5a, b).

We established a two-colored single-molecule co-localization assay to quantify the MAVS recruitment on a shorter dsRNA substrate (1000 bp, Fig. 4e; Supplementary information, Fig. S5c). In the absence of LGP2, frequent MDA5 binding events were detected, characterized by a temporary increase followed by a decrease in MDA5 fluorescence intensity, consistent with dynamic assembly and disassembly of ATM clusters (–LGP2, Fig. 4f, g). Upon addition of LGP2, stable microfilaments rapidly formed and remained bound to the substrate (+LGP2, Fig. 4f, g). While the peak MDA5 intensity increased with elevated LGP2 protein concentration, recruitment of MAVS-CARD was strictly dependent on LGP2 (Fig. 4h, i). These results suggest that MDA5–LGP2 microfilaments, but not ATM clusters, support MDA5–MAVS interactions, and that a crucial role of LGP2 is to promote microfilament formation, thereby facilitating the assembly of CARDs into functional oligomers for activation.

We stimulated *RIG-I* knockout HEK293 cells (293∆RIG-I) with dsRNA of various lengths and measured interferon mRNA levels to further explore the roles of 1D movement in cellular MDA5 activation (Supplementary information, Fig. S5d, e). dsRNA ligands shorter than 1 kb did not effectively induce interferon expression through endogenous RLRs, whereas stimulation with longer dsRNA resulted in increased interferon production (endogenous RLRs, Fig. 4j). Overexpressing MDA5 in 293∆RIG-I did not notably enhance signaling activity with any dsRNA ligands (MDA5, Fig. 4j), indicating that cellular MDA5 did not spontaneous form active filaments on these dsRNA ligands, even at much higher protein concentrations (Supplementary information, Fig. S5f). By contrast, the simultaneous overexpression of MDA5 and LGP2 largely boosted interferon production, consistent with the notion that LGP2 efficiently captures MDA5 motors on dsRNA (MDA5 + LGP2, Fig. 4j; Supplementary information, Fig. S5g).

### 1D movement prevents abnormal MDA5 activation

**Fig. 5:**
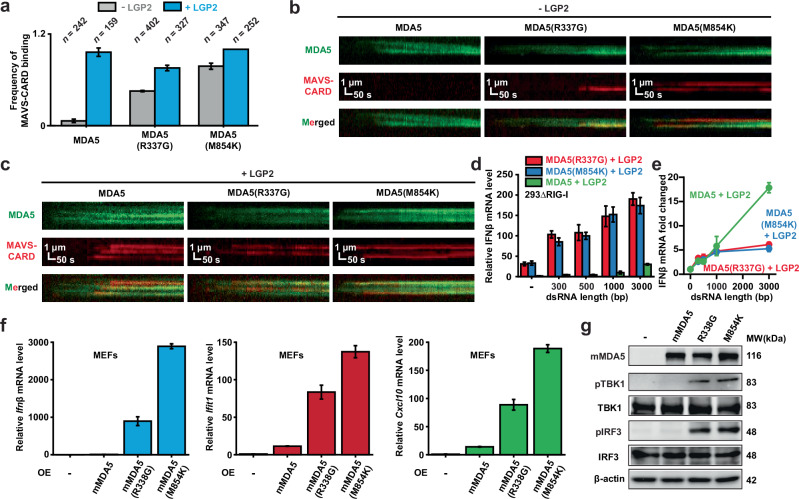
1D movement protects MDA5 from abnormal activation. **a** The frequency of MAVS-CARD recruitment by 100 nM MDA5, MDA5(R337G) and MDA5(M854K) in the absence or presence of 100 nM LGP2 (mean ± SD; *n* = number of dsRNA molecules). **b** Representative kymographs showing the recruitments of AF647-MAVS-CARD (100 nM) by Cy3-MDA5, Cy3-MDA5(R337G) and Cy3-MDA5(M854K) (100 nM) in the absence of LGP2. Cy3-MDA5 protein is shown in green and AF647-MAVS-CARD is shown in red. **c** Representative kymographs showing the recruitments of AF647-MAVS-CARD (100 nM) by Cy3-MDA5, Cy3-MDA5(R337G) and Cy3-MDA5(M854K) (100 nM) in the presence of LGP2 (100 nM). Cy3-MDA5 protein is shown in green and AF647-MAVS-CARD is shown in red. **d** Column plots of relative IFNβ mRNA levels showing the MDA5 signaling activity under various conditions. 293∆RIG-I with MDA5(R337G) and LGP2 co-overexpression (MDA5(R337G)+LGP2), 293∆RIG-I with MDA5(M854K) and LGP2 co-overexpression (MDA5(M854K)+LGP2) and 293∆RIG-I with MDA5 and LGP2 co-overexpression (MDA5+LGP2) were stimulated with dsRNA substrates of different lengths. **e** Relative IFNβ mRNA levels in relation to dsRNA length. **f** Column plots of relative *Ifnβ*, *Ifit1* and *Cxcl10* mRNA levels showing the MDA5 signaling activity in MEFs under various conditions. **g** Immunoblotting showing the phosphorylation of TBK1 and IRF3 in MEFs cells. All single-molecule studies were performed at least two separate times. All cell-based assays were repeated independently at three times with similar results. Error bars represent SEM between measurements, centered on the mean of a single experiment.

One might envision that ATPase deficiency prevents MDA5 from translocating, thereby bypassing the requirement for LGP2 and spontaneously assembling into filaments. To test this hypothesis, we measured MAVS-CARD recruitment by MDA5, MDA5(R337G), and MDA5(M854K) on 11.6-kb dsRNA in the absence of LGP2 (Fig. 5a, b). Under the conditions allowing ATP hydrolysis, abundant ATM clusters were formed by wild-type MDA5 but failed to recruit MAVS-CARD (Fig. 5a, b; Supplementary information, Fig. S6a). By contrast, both MDA5(R337G) and MDA5(M854K) supported numerous MAVS-CARD binding events, indicating that these ATPase-deficient mutants spontaneously formed filaments and their CARDs had assembled into functional oligomers (Fig. 5a, b; Supplementary information, Fig. S6a). Although LGP2 enhanced MAVS-CARD recruitment for all MDA5 variants, it was strictly required only for wild-type MDA5 (Fig. 5a, c; Supplementary information, Fig. S6a). These findings suggest that mobile and immobile MDA5 assemble into filaments through distinct pathways, with LGP2 essential for the mobile form but dispensable for the immobile form.

Co-expression of LGP2 and ATPase-deficient MDA5, combined with dsRNA stimulation in 293ΔRIG-I cells, led to a substantial increase in interferon levels (Fig. 5d; Supplementary information, Fig. S6b). Signaling remained consistently high across dsRNA of varying lengths, suggesting that MDA5(R337G) and MDA5(M854K) substantially lose their capacity for length-dependent discrimination. This impaired discrimination was independent of LGP2, as overexpression of ATPase-deficient MDA5 alone yielded similar results (Supplementary information, Fig. S6c, d), consistent with previous studies showing that ATPase-deficient MDA5 was readily activated by short dsRNA.^18^ By contrast, substituting wild-type MDA5 for the ATPase-deficient variants markedly reduced interferon production and robustly sharpen the length-dependent discrimination (Fig. 5d, e). These observations suggest that filament assembly by mobile MDA5 requires LGP2 and responds to dsRNA length, whereas immobile MDA5 assembles with little sensitivity to length.

In mouse embryonic fibroblasts (MEFs), overexpression of ATPase-deficient mMDA5 mutants (R338G and M854K, corresponding to human R337G and M854K), but not the wild-type protein, led to elevated expression of interferon and ISGs, along with increased phosphorylation of TBK1 and IRF3, even in the absence of dsRNA stimulation, mimicking an autoimmune-like state (Fig. 5f, g). These findings further demonstrate that ATP-hydrolysis-driven 1D motion is essential for preventing aberrant MDA5 activation, and that this regulatory mechanism is conserved across species.

## Discussion

**Fig. 6:**
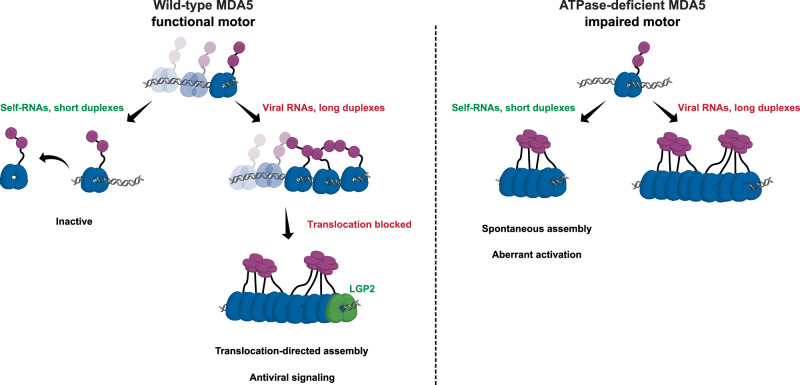
Distinct pathways of filament assembly by wild-type and ATPase-deficient MDA5 on dsRNA. Left: wild-type MDA5 uses ATP hydrolysis to translocate along dsRNA. On self-RNAs, the motors dissociate quickly and fail to assemble into filaments. On viral RNAs, MDA5 forms ATM clusters with spacing between individual molecules. LGP2 binding serves as a roadblock, compressing MDA5 within these clusters and promoting microfilament formation. This translocation-driven assembly proceeds efficiently on long dsRNA duplexes, supporting antiviral signaling. Right: ATPase-deficient MDA5, which lacks 1D translocation activity, assembles into filaments spontaneously on dsRNA, regardless of duplex length. This indiscriminate filament formation causes aberrant activation on self-RNAs.

The prevailing model suggests that multiple MDA5 molecules assemble into filaments through helicase–helicase stacking, with ATP hydrolysis proposed to drive filament disassembly from the ends^26,28^ or to couple to changes in helical twist.^53^ Although ATP-hydrolysis-driven 1D translocation was initially speculated, it was later considered unfavorable,^53^ likely due to limited dynamic studies or insufficient spatial resolution.^28^ Here, we provide direct evidence that MDA5 undergoes 1D translocation along dsRNA (Fig. 1), and propose that immobile and mobile MDA5 form filaments through two distinct pathways (Fig. 6). Without 1D motion, immobile MDA5 may remain bound to dsRNA, spontaneously assemble into filaments (Figs. 5b, d and 6). By contrast, long-range movement on dsRNA enables mobile MDA5 to form ATM clusters with spacing between them, and subsequent LGP2 binding likely act as a roadblock to compress these motors and promote microfilament formation (Fig. 6). Although how LGP2 facilitates efficient filament assembly remains to be fully defined, including the required dsRNA length, the number of MDA5 molecules involved, and the stoichiometry between MDA5 and LGP2, the filament-forming interface of MDA5 may contribute to microfilament assembly, as a single LGP2 potentially arrests multiple MDA5 motors (Fig. 6). In addition, accurately determining the spacing between MDA5 motors within an ATM cluster remains challenging due to the diffraction limit and may require super-resolution imaging.

It has been proposed that MDA5 is capable of measuring the length of a dsRNA molecule by ATP hydrolysis.^1,54,55^ The dissociation of MDA5 motor at a dsRNA end suggests that this 1D movement may scan the nucleic acid backbone for discontinuities, which, upon encounter, trigger a conformational change in the helicase domain that opens the ring-like structure (Fig. 1c, i). As a result, the efficiency of translocation-directed filament assembly likely depends on dsRNA length, either because MDA5 dissociates too rapidly from short dsRNA to be captured by LGP2, or because the restricted movement on short duplexes fails to promote the cooperative binding required for effective filament formation (Fig. 6). Immobile MDA5, such as ATPase mutants, may also function as a roadblock to mobile MDA5 motors; however, their filament assembly can occur spontaneously at high protein concentrations, does not require long-range movement, and is insensitive to dsRNA length (Figs. 5e and 6). RNA secondary structures, such as internal loops, bulges, or pseudoknots, commonly found in self-RNAs may potentially serve as discontinuities that disrupt MDA5 translocation and contribute to the discrimination between self- and nonself-RNAs. While single-molecule and cellular studies here provide evidence for the role of MDA5 translocation, future validation in primary immune cells or animal models will be important, particularly for those gain-of-function mutations that could potentially impair motor activity independently of ATPase function.

The helicase domains and CTDs of RLR proteins exhibit similar sequence conservation,^56^ however, they may play different roles in downstream signaling. Previous studies demonstrated that RIG-I also functions as an ATP-dependent motor after recognizing a dsRNA end containing 5’-ppp.^39,57,58^ While MDA5 translocates along long dsRNA to suppress spontaneous filament formation and promote translocation-directed filament assembly, the physiological function of RIG-I translocation remains unclear and requires further investigation. LGP2, the third member of the RLR family, does not operate as efficiently as an MDA5 motor on long dsRNA (Fig. 3c), however, we cannot rule out the possibility of LGP2 performing translocation for a short distance on dsRNA that is unable to be detected in our system.^41^ Moreover, TRIM65-mediated polyubiquitination and ZCCHC3 may regulate MDA5’s 1D movement, stabilize the filament and CARD tetramer in cells, and promote delivery to MAVS.^11,25,59–61^

RLRs are not the only sensors capable of moving along dsRNA. Many dsRNA-binding proteins (dsRBPs), including protein kinase R (PKR), oligoadenylate synthases (OASes), toll-like receptor 3 (TLR3), and NOD-, LRR-, and pyrin domain-containing 1 (NLRP1), also respond to dsRNA in a length-dependent manner.^62^ Some of the dsRBPs have been directly observed diffusing along dsRNA in an ATP-independent manner, suggesting that 1D movement might be commonly involved in immune signaling.^40^ Overall, our findings reveal that protein motions on nucleic acids significantly influence oligomer assembly, establishing an indispensable role of 1D motion in maintaining immune homeostasis.

## Materials and Methods

### Cell culture and dsRNA stimulation assay

HEK293(ATCC), HEK293T(ATCC), and MEF (provided by Dr. Fajian Hou) were cultured in Dulbecco’s modified Eagle’s medium (DMEM, Thermo Fisher Scientific) supplemented with 10% fetal bovine serum (Thermo Fisher Scientific), and 100 U/mL of penicillin/streptomycin (Thermo Fisher Scientific). A549 (ATCC) cells were cultured in Ham’s F-12K (Kaighn’s) medium (Thermo Fisher Scientific) supplemented with 10% FBS, 1x GlutaMax (Thermo Fisher Scientific,) and 100 U/mL of penicillin/streptomycin (Thermo Fisher Scientific). All cells were cultured at 37 °C with 5% CO_2_ and tested for mycoplasma before use. Transfections were carried out using Lipofectamine 2000 Reagent (Thermo Fisher Scientific) according to the manufacturer’s protocols. For dsRNA stimulation assay in 293∆RIG-I, cells were seeded in a 12-well plate and transfected with 500 ng of the pCMV-Myc-MDA5, MDA5(R337G), MDA5(M854K) and/or pCMV-LGP2-3× Flag plasmids, respectively. After 24 h, cells were transfected with 0.5 μg/mL dsRNAs and incubated for 24 h.

### Generation of gene knockouts cell line using CRISPR-Cas9

To generate *MDA5* or *LGP2* knockout A549, cells were initially seeded in a 6-well plate and subsequently transfected with 2 μg of either the pC338-MDA5 sgRNA or pC338-LGP2 sgRNA plasmids, respectively. Following incubation for 36 h, the medium was replaced, and 2 μg/mL of puromycin was introduced for selection. After an additional 48 h, single cells were seeded into a 96-well plate and expanded for screening through sequencing and immunoblotting. To verify the knockouts of A549 cells, wild-type A549, ΔMDA5, and ΔLGP2 cells were initially seeded in a 6-well plate and 5 ng/mL of IFNβ (Peprotech) was introduced. After 24 h incubation, cells were collected for immunoblotting.

To generate 293∆RIG-I, HEK293T cells were initially seeded in a 6-well plate and subsequently transfected with 1.5 μg of psPAX2 plasmid, 1 μg of pMD2.G plasmid, and 2 μg of lenti-Cas9-sgRNAs using polyethylenimine (Polysciences). Culture supernatants containing lentiviruses were harvested at 48 h post-transfection, passed through a 0.45-μm pore size filter, and used to infect HEK293 cells. Transduced cells were selected in medium containing 1 μg/mL of puromycin for 2 days. The *RIG-I* knockout efficiency was verified by western blot analysis followed by 5 ng/mL of IFNβ treatment (Peprotech) for 48 h.

### Lentivirus-mediated LGP2 complementation

GFP, LGP2 and LGP2(intermut) were inserted into pLVX-IRES-puro digested with *EcoR*I and *Xba*I, respectively. To produce lentiviral particles, HEK293T cells were co-transfected with pLVX, psPAX2, and MD2.G at a 5:3:2 ratio using Lipofectamine 2000. The medium was replaced 24 h post-transfection, and supernatants were harvested after 72 h. The supernatants were subsequently centrifuged at 3000 × *g* for 5 min at 4 °C and filtered through a 0.45-μm filter to remove cellular debris. Cells stably expressing GFP, LGP2, or LGP2(intermut) were generated by retroviral transduction and selected with puromycin (2 μg/mL). Protein expression was verified by western blot analysis.

### EMCV infection assay

Wild-type A549, ΔMDA5, ΔLGP2, and ΔLGP2 cells complemented with LGP2 were seeded in a 48-well plate, followed by the addition of EMCV (1 MOI) into the culture medium. Total RNA was then collected at 12 h or 24 h post-infection for further analysis.

### Antibodies

The antibodies used for immunoblots were: anti-RIG-I (Cell Signaling Technology, Cat# 3743S), anti-MDA5 (ProteinTech, Cat# 21775-1-AP), anti-LGP2 (ProteinTech, Cat# 11355-1-AP), anti-β-actin-HRP (Cell Signaling Technology, Cat# 12262S), anti-rabbit-IgG-HRP (Cell Signaling Technology, Cat# 7074S) and anti-GFP (ProteinTech, Cat# 50430-2-AP), anti-TBK1 (Cell Signaling Technology, Cat# 3504S), anti-Phospho-TBK1 (Cell Signaling Technology, Cat# 5483S), anti-IRF3 (ProteinTech, Cat# 11312-1-AP), anti-Phospho-IRF3 (Thermo Fisher Scientific, Cat# PA5-143363).

### RT-qPCR quantification and statistical analysis

Total RNAs were extracted using RNAiso Plus reagent (Takara) and cDNA was synthesized using PrimeScript™ RT reagent Kit with gDNA Eraser (Perfect Real Time) (Takara) according to the manufacturer’s instructions. Interferon and ISGs expressions were measured using TB Green® Premix Ex Taq™ (Tli RNaseH Plus) reagent (Takara) on a QuantStudio™ 6 Flex (Applied Biosystems), where β-actin was examined as an internal control for normalization. Primers for RT-qPCR were listed in Supplementary information, Table S1. All qPCR experiments were repeated independently at three times.

### Plasmid construction, recombinant protein labeling and purification

The human MDA5, MDA5(Q57E), MDA5(R337G), MDA5(M854K), MDA5∆N (residues 287–1025), LGP2 and LGP2(intermut) proteins were labeled using sortase-mediated peptide ligation.^63,64^ The RLR genes were amplified by PCR (Supplementary information, Table S1), digested with *BamH*I and *Kpn*I, and inserted into pFastbac1 baculovirus expression plasmid. Hexa-histidine (his_6_) and sortase recognition sequence (srt, LPETG) were introduced onto the N-terminus of MDA5 protein, or C-terminus of LGP2 protein. For cellular overexpression plasmids, MDA5 was inserted into pCMV-N-Myc digested with *EcoR*I, and LGP2 was inserted into pCMV-C-3× Flag, digested with *EcoR*I. The MDA5(Q57E), MDA5(R337G), MDA5(M854K) and LGP2(intermut) mutations were generated using the KOD-Plus-Mutagenesis Kit (Toyobo). Two serine residues separated the his_6_ and srt, and these tags were separated from the RLR proteins by two or four glycine residues. All the plasmid constructs were amplified in *E. coli* DH5α and verified by DNA sequencing.

The human MDA5 and LGP2 proteins were overexpressed in SF9 insect cells using the Bac-to-Bac Baculovirus Expression System (Thermo Fisher Scientific). All purification procedures were carried out at 4 °C. SF9 cells were harvested and suspended in Lysis Buffer (25 mM Tris-HCl pH 7.5, 500 mM NaCl, 20% glycerol, and 20 mM imidazole). Cells were thawed on ice, passed through a 25 G needle five times, and centrifuged at 50,000× *g* for 1 h. The supernatant was loaded onto a Ni-NTA (Qiagen) column, washed with Buffer A (25 mM Tris-HCl pH 7.5, 500 mM NaCl, 20% glycerol and 20 mM imidazole), and eluted with Buffer B (25 mM Tris-HCl pH 7.5, 500 mM NaCl, 20% glycerol, and 200 mM imidazole). Peak fractions containing recombinant proteins were pooled and dialyzed against Labeling Buffer (50 mM Tris-HCl pH 7.5, 150 mM or 500 mM NaCl, and 20% glycerol). To label MDA5, recombinant proteins were incubated with an improved version of sortase^65^ and peptides (Cy3/Cy5-CLPETGG, purchased from ChinaPeptides Co., LTD) as described^43^ (protein: sortase: peptide in the molar ratio of 1:2:5). To label LGP2, recombinant proteins were incubated with sortase and peptides (GGGC-Cy3/Cy5, purchased from ChinaPeptides Co., LTD). The reaction was incubated for 1 h on ice and loaded onto a Ni-NTA column. Flow-through proteins were collected and diluted with Buffer C (25 mM Tris-HCl pH 7.5, 1 mM DTT, 20% glycerol and 0.1 mM EDTA) to reduce NaCl concentration within the buffer (~100 mM). The proteins were then loaded onto a heparin column, washed extensively with Buffer C plus 100 mM NaCl and eluted with Buffer C plus 1 M NaCl. For MDA5, further contaminants were separated by size-exclusion chromatography (Superdex 200 10/300 GL, GE Healthcare). Protein-containing fractions were dialyzed against Storage Buffer (25 mM Tris-HCl pH 7.5, 150 mM or 500 mM NaCl, 1 mM DTT, 0.1 mM EDTA, and 20% glycerol) and frozen at –80 °C.

A MAVS-CARD protein containing three mutations (D23K, E26K, and E80K) was used for single-molecule imaging. These mutations have been reported to increase protein solubility without disrupting MDA5-MAVS interactions.^11,66^ A pET47b plasmid encoding MAVS-CARD (residues 1–97)^67^ with an N-terminal His-tag and a C-terminal SNAP-tag was a gift from Sun Hur lab. The MAVS-CARD(D23K/E26K/E80K) (referred to as MAVS-CARD in the following text and main text) was generated using the KOD-Plus-Mutagenesis Kit. After transformation with the MAVS-CARD expression plasmid, a single colony of BL21(DE3) cells was diluted into 1 L of LB containing 50 μg/mL kanamycin. At OD_600_ = 0.4, the growth temperature was decreased to 18 °C and the expression protein was induced by the addition of IPTG (0.2 mM) at 18 °C for 16 h. Cells were collected and resuspended in Freezing Buffer (25 mM Tris-HCl pH 7.5, 500 mM NaCl, 20% glycerol and 20 mM imidazole). Cell pellets were freeze-thawed three times, sonicated twice, and centrifuged at 50,000× *g* for 1 h. The supernatants were then loaded onto a Ni-NTA column, washed with Buffer A, Buffer D (25 mM Tris-HCl pH 7.5, 500 mM NaCl, 20% glycerol, and 60 mM imidazole), and eluted with Buffer B. Fractions containing MAVS-CARD were pooled, and incubated with SNAP-Surface Alexa Fluor 647 substrate (New England Biolabs) in 1 mM DTT at 4 °C for overnight. After labeling, the protein was diluted with Buffer E (25 mM Tris-HCl pH 7.5, 500 mM NaCl, 20% glycerol) to reduce imidazole concentration within the buffer (~20 mM). Proteins were then loaded onto the Ni-NTA column, washed with Buffer A and eluted with Buffer B. Protein-containing fractions were dialyzed against Storage Buffer and frozen at –80 °C. Given the observations that purified MAVS-CARD proteins exist in short filamentous structures, a denaturing-refolding approach was employed to obtain monomeric MAVS-CARD as described.^67^ In brief, MAVS-CARD was denatured in 6 M guanidinium hydrochloride (GuHCl) at 37 °C for 30 min, followed by dialysis in Refolding Buffer (20 mM Tris-HCl, pH 7.5, 500 mM NaCl, 0.5 mM EDTA, and 2 mM DTT) at 4 °C for 1 h, and was immediately used for single-molecule imaging.

The concentrations and labeling efficiencies of all recombinant proteins were determined by measuring protein absorbance at 280 nm and Cy3/Cy5 absorbance at 550/650 nm, respectively (Supplementary information, Table S2).

### ATPase analysis

The ATPase activities of MDA5 and LGP2 were measured utilizing an ATPase/GTPase Activity Assay Kit (Sigma). The analysis was carried out with 200 nM protein and 100 ng RNA substrate (for ssRNA: ssRNA31 (Supplementary information, Table S1); for dsRNA: 11.6-kb dsRNA) in a 40 μL reaction mixture comprised of 20 mM Tris-HCl, pH 7.5, 150 mM NaCl, 1 mM ATP, 5 mM MgCl_2_, and 1 mM DTT. For MDA5, the reactions were performed at 23 °C for 0, 1, 2, 3, and 4 min followed by quenching with 200 μL of malachite green reagents. For LGP2, the reactions were performed at 23 °C for 0, 10, 20, 30, and 40 min followed by quenching with 200 μL of malachite green reagents. Samples were incubated at 23 °C for additional 30 min. Free phosphate was determined by measuring absorbance at 620 nm using a microplate reader Eon (Bio Tek). Data were fit to a linear function to calculate the rates of ATP hydrolysis and the turnover numbers (k_cat_).

### Negative stain EM

To visualize MDA5 filaments, 250 nM of either wild-type or mutant MDA5 was incubated with 3.5 µg/mL 1-kb dsRNA in a buffer containing 20 mM Tris-HCl, pH 7.5, 150 mM NaCl, 5 mM MgCl_2_, 1 mM DTT, and 5 mM ATP (or ADPCP) at room temperature for 20 min. Samples were spotted on glow-discharged carbon-coated grids and stained with uranyl acetate (2% (wt/vol)). Images were collected using a FEI Tecnai G2 Spirit 120 KV transmission electron microscope at 67,000× magnification. To visualize MDA5-LGP2 filaments, 75 nM MDA5, 75 nM LGP2 and 3.5 µg/mL 1-kb dsRNA were incubated in 20 mM Tris-HCl, pH 7.5, 150 mM NaCl, 5 mM MgCl_2_, 1 mM DTT, and 5 mM ATP at room temperature for 20 min. Samples were spotted on glow-discharged carbon-coated grids and stained with uranyl acetate (2% (wt/vol)). Images were collected using a FEI Tecnai G2 Spirit 120 KV transmission electron microscope at 110,000× magnification.

### Preparation of 11.6-kb dsRNA

Two complementary 11.6-nt ssRNA strands were produced by SP6 in vitro transcription, followed by annealing together along with two biotin-labeled ssRNA linkers to generate an 11.6-kb dsRNA substrate (Supplementary information, Fig. S1a). In brief, two plasmids containing the SP6 promoter and sense/anti-sense strand sequences were constructed, amplified in *E. coli* DH5α, and verified by DNA sequencing. DNA templates were then linearized by *Kpn*I (for sense strand) or *BamH*I (for anti-sense strand) digestion, and purified by phenol-chloroform extraction and ethanol precipitation. RNAs were transcribed in vitro using SP6 RNA Polymerase (Thermo Fisher Scientific), followed by phenol-chloroform extraction and ethanol precipitation. Sense and anti-sense RNA strands (100 nM for each), along with RNA Linker 1 and RNA Linker 2 (10 µM for each; Supplementary information, Table S1) were mixed in Annealing Buffer (20 mM Tris-HCl pH 7.5, 50 mM NaCl, 1 mM EDTA), denatured at 95 °C for 10 min, then cooled down slowly to 23 °C. The resulting product was separated on a 0.75% agarose gel and the 11.6-kb dsRNA was excised and purified using Agarose Gel DNA Extraction Kit (Takara).

For dsRNA substrates used in two-colored single-molecule co-localization assay, two complementary ssRNA strands were produced by T7 or SP6 in vitro transcription, followed by annealing together along with RNA Linker 3 (Supplementary information, Table S1) to generate dsRNAs of varying lengths. Briefly, DNA templates of different lengths containing SP6 and T7 promoters were obtained by PCR and purified from agarose gel. Sense and antisense RNAs were transcribed in vitro and annealed with RNA Linker 3. The resulting product was separated on a 0.75% agarose gel and the dsRNA substrates were excised and purified using the Agarose Gel DNA Extraction Kit (Takara).

### Single-molecule imaging buffers and experiment conditions

The single-molecule Imaging Buffer contains 20 mM Tris-HCl, pH 7.5, 2 mM DTT, 0.2 mg/mL acetylated BSA (Molecular Cloning Laboratories), 0.0025% P-20 surfactant (GE healthcare), 2 mM ATP (unless stated otherwise), 1.5 mM MgCl_2_, and 100 mM NaCl. All single-molecule experiments were carried out at 23 °C.

### smTIRF microscopy

All the smTIRF data were acquired on a custom-built prism-type TIRF microscope established on the Olympus microscope body IX73.^43^ Fluorophores were excited using the 532 nm for green and 637 nm for red laser lines built into the smTIRF system. Image acquisition was performed using an EMCCD camera (iXon Ultra 897, Andor) after splitting emissions by an optical setup (OptoSplit II emission image splitter, Cairn Research). Micro-Manager image capture software was used to control the opening and closing of a shutter, which in turn controlled the laser excitation.

The 11.6-kb dsRNA (50 pM) in 500 μL T50 buffer (20 mM Tris- HCl, pH 7.5, 50 mM NaCl) was injected into a custom-made flow cell chamber and stretched by laminar flow (300 μL/min). The stretched dsRNA was anchored at both ends onto a neutravidin-coated, PEG-passivated quartz slide surface, and the unbound dsRNA was flushed by similar laminar flow. The flow cell has dimensions of 35 mm × 6 mm × 0.11 mm, corresponding to a cross-sectional area of 0.66 mm². The average length (*L*) of the 11.6-kb dsRNA was measured to be 2.5 μm, the contour length (*L*_*C*_) was 3.3 μm, and the relative elongation ratio (*L/L*_*C*_) was 0.76. Based on the dsRNA force-extension curve, the stretching force was estimated to be less than 1 pN.^68^

Unless stated otherwise, a 5000-ms frame rate with 300-ms laser exposure time was used to minimize photo-bleaching during single-molecule imaging. The dsRNA was located by staining with SYBR Gold (0.2 X, Invitrogen) after real-time recording.

To examine MDA5 translocation on dsRNA, 3 nM Cy3-MDA5 in Imaging Buffer was introduced into the flow cell chamber. The MDA5 molecules on dsRNA were then monitored in real-time for 25 min in the absence of flow. To examine the formation of MDA5 ATM cluster, 15 nM Cy3-MDA5 in Imaging Buffer was introduced into the flow cell chamber. The ATM clusters on dsRNA were then monitored in real-time for 25 min in the absence of flow.

To investigate MDA5 synchronization during ATP hydrolysis, 15 nM Cy3-MDA5 with ADPCP or 60 nM Cy3-MDA5 without ATP in Imaging Buffer were first introduced into the flow cell chamber. After 17 min incubation, the flow cell was flushed with 2 mM ATP in Imaging Buffer, and the MDA5 on dsRNA were monitored in real-time for 21 min in the absence of flow.

To examine the collision of MDA5, 15 nM or 100 nM Cy3-MDA5 in Imaging Buffer was introduced into the flow cell chamber. To examine the individual motors within colliding foci, 3 nM Cy3-MDA5 and 97 nM unlabled-MDA5 in Imaging Buffer were introduced into the flow cell chamber. The MDA5 on dsRNA was then monitored in real-time for 25 min in the absence of flow.

To examine spontaneous filament assembly with ATPase-deficient MDA5, 100 nM Cy3-MDA5(R337G) or MDA5(M854K) in Imaging Buffer with or without ATP was introduced into the flow cell chamber. The MDA5 on dsRNA was then monitored in real-time for 25 min in the absence of flow.

To examine LGP2 binding on dsRNA, 60 nM Cy3-LGP2 with or without 60 nM unlabeled MDA5, MDA5(R337G), or MDA5(M854K) in Imaging Buffer was introduced into the flow cell chamber. The LGP2 on dsRNA was then monitored in real-time for 25 min in the absence of flow. To examine the MDA5–LGP2 interactions on dsRNA, 3 nM Cy3-MDA5 and 3 nM Cy5-LGP2, 0.5 nM Cy3-MDA5∆N and 0.5 nM Cy5-LGP2, or 30 nM Cy3-MDA5(R337G)/Cy3-MDA5(M854K) and 30 nM Cy5-LGP2 proteins in Imaging Buffer were introduced into the flow cell chamber and protein-protein interactions monitored in real-time for 25 min in the absence of flow.

To examine the interactions between MDA5–LGP2 microfilaments and MAVS-CARD, Cy3-MDA5 (20 nM), unlabeled LGP2 (20 nM) and AF647-MAVS-CARD (100 nM) in Imaging Buffer were introduced into the flow cell chamber. To examine the interactions between MDA5 and MAVS-CARD, Cy3-MDA5 (100 nM), Cy3-MDA5(R337G) (100 nM) or Cy3-MDA5(M854K) (100 nM) and AF647-MAVS-CARD (100 nM) in Imaging Buffer with or without unlabeled LGP2 (100 nM) were introduced into the flow cell chamber and protein–protein interactions were monitored in real-time for 17 min in the absence of flow.

To examine MDA5 binding to 1000-bp dsRNA substrate in two-colored single-molecule co-localization assay, a 3000-ms frame rate with 300-ms laser exposure time was used in single-molecule imaging. Cy5-labeled dsRNA (50 pM) in 500 μL T50 buffer was injected into a custom-made flow cell chamber, and incubated for 3 min. 15 nM Cy3-MDA5 in Imaging Buffer was introduced into the flow cell chamber, the MDA5 molecules on dsRNA were then monitored in real-time for 30 min in the absence of flow.

To examine the interactions between MDA5 and MAVS-CARD on 1000-bp dsRNA substrate, Cy5-labeled dsRNA was introduced into the flow cell chamber. A photobleaching step was then carried out to remove the Cy5 signals from the dsRNA, allowing for the recording of the dsRNA’s position. Cy3-MDA5 (15 nM), unlabeled LGP2 (0 nM, 3 nM, 5 nM, 10 nM, and 15 nM), and AF647-MAVS-CARD (100 nM) in Imaging Buffer were then introduced into the flow cell chamber and protein-protein interactions were monitored in real-time for 30 min in the absence of flow.

### Position determination on dsRNA

To determine the positions of MDA5 on RNA, the 11.6-kb dsRNA was stained with SYBR Gold (0.2 X, Invitrogen). The left (*P*_*L*_) and the right (*P*_*R*_) end positions of the dsRNA were determined by plotting the fluorescent intensities along the length of the stained RNA as previously described.^35^ Horizontal positions of protein particles (*P*_*P*_) on dsRNA were tracked by DiaTrack 3.05 (Sydney, Australia), where the particle intensities were fit to a two-dimensional Gaussian function to obtain their positions with sub-pixel resolution. The positions were then converted to lengths in bp by the following equation: 11,645 bp × (*P*_*P*_−*P*_*L*_) / (*P*_*R*_−*P*_*L*_), in which 11,645 bp is the length of the dsRNA. A 1000-bp (~1 pixels) binning size was used to construct the position histograms.

### Data analysis of TIRF imaging

All kymographs were generated along the dsRNA by a kymograph plugin in ImageJ (J. Rietdorf and A. Seitz, EMBL Heidelberg). For studies involving Cy3-MDA5 and Cy5-LGP2, or Cy3-MDA5 and AF647-MAVS-CARD, fluorescent molecules in two channels were co-localized using a custom-written MATLAB script. Particles were tracked using DiaTrack 3.05 to obtain single-molecule fluorescent intensities and trajectories.

To determine the frequency of MDA5 translocation on dsRNA, single-molecule movies were recorded for 25 min and the translocating MDA5 molecules with a minimum lifetime of 40 s were counted as the number of MDA5 translocation (*N*_MDA5-trans_). To determine the frequency of MDA5 ATM cluster, single-molecule movies were recorded for 25 min. The MDA5 molecules with increased fluorescent intensities (>10-fold of the initial intensity of a single MDA5) during 1D translocation were counted as the number of MDA5 ATM clusters (*N*_MDA5-ATM cluster_).

To determine the frequency of filament-like MDA5, single-molecule movies were recorded for 25 min. The MDA5-coated dsRNA molecules with a minimum length of 7 pixels were counted as the number of MDA5 filament-like structure (*N*_Filament-like_).

To determine the frequency of MDA5 ATM cluster, single-molecule movies were recorded for 25 min. The MDA5 molecules with increased fluorescent intensities (> 10-fold of the initial intensity of a single MDA5) during 1D translocation were counted as the number of mobile MDA5 ATM cluster (*N*_ATM-mobile_). In the presence of LGP2, the immobile MDA5 molecules with increased fluorescent intensities ( > 10-fold of the initial intensity of a single MDA5) were counted as the number of immobile MDA5 ATM cluster (*N*_ATM-immobile_). To determine the distribution of ATM cluster collision, single-molecule movies were recorded for 25 min. An event where two ATM clusters move in opposite directions and then collide was considered a head-on collision.

To determine the proportion distribution of MDA5∆N translocation and stop events, single-molecule movies were recorded for 25 min and the translocating MDA5∆N molecules with a minimum lifetime of 40 s were counted as the number of mobile MDA5∆N events (*N*_MDA5∆N-mobile_). The MDA5∆N molecules undergoing 1D translocation and subsequently transitioning into immobile state were counted as the number of immobile MDA5∆N events (*N*_MDA5∆N-immobile_).

To determine the frequency of MAVS-CARD binding, single-molecule movies were recorded for 17 min. dsRNA molecules exhibiting one or more MAVS-CARD binding events lasting at least 50 s were counted as MAVS-CARD-bound molecules (*N*_MAVS-CARD_).

Following the real-time single-molecule recording, the number of dsRNA molecules (*N*_RNA_) was determined by SYBR Gold staining. The frequencies of MDA5 translocation (*F*_MDA5-trans_), MDA5 ATM cluster (*N*_MDA5-ATM cluster_), MDA5 filament-like structure (*N*_Filament-like_), mobile MDA5 ATM cluster (*N*_ATM-mobile_), immobile MDA5 ATM cluster (*N*_ATM-immobile_), and MAVS-CARD binding (*F*_MAVS-CARD_) were calculated using the following equations that also included corrections for labeling efficiencies of the proteins (the numbers in the denominator, Supplementary information, Table S2):

$${F}_{{{\rm{MDA5-trans}}}}=\frac{{N}_{{{\rm{MDA5-trans}}}}}{{N}_{{{\rm{RNA}}}}\times 0.50} \quad \qquad {F}_{{{\rm{MDA}}}5-{{\rm{ATM\; cluster}}}}=\frac{{N}_{{{\rm{MDA}}}5-{{\rm{ATM\; cluster}}}}}{{N}_{{{\rm{RNA}}}}}$$

$${{F}}_{{{\rm{Filament-like}}}}=\frac{{N}_{{{\rm{Filament-like}}}}}{N_{{\rm{RNA}}}}$$

$${F_{{\rm{ATM-immobile}}}}=\frac{{N_{{\rm{ATM-immobile}}}}}{{N}_{{\rm{RNA}}}}\qquad \quad {F_{{\rm{ATM-mobile}}}}=\frac{{N_{{{\rm{ATM-mobile}}}}}}{{N_{{\rm{RNA}}}}}$$

$${F}_{{{\rm{MAVS}}}-{{\rm{CARD}}}}=\frac{{N}_{{\rm{MAVS-CARD}}}}{{N}_{{{\rm{RNA}}}}}$$

All single-molecule frequency studies were performed at least two separate times.

### Survival probability analysis

To plot the survival probability of a single MDA5 motor and a single MDA5 motor within mobile foci, all events were synchronized in time, with the initial event count set to 1. MDA5 motor dissociation was quantified in 180-s time bins. To plot the survival probability of MDA5∆N molecules on dsRNA with or without LGP2, all events were synchronized in time, with the initial event count set to 1. MDA5∆N dissociation was quantified in 240-s time bins.

### Translocation rates analysis

The association (*P*_*A*_) and dissociation (*P*_*D*_) positions of MDA5 translocation were determined by particle trajectories on dsRNA. The time intervals (*T*) between association and dissociation events were extracted from kymograph. Translocation rates (*R*) were calculated using the following equation: 11,645 bp × (*P*_*A*_ − *P*_*D*_) / (*T* × 2.5 µm), where 2.5 µm is the average length of dsRNA observed by smTIRF microscopy (Fig. 1b). Absolute values were used to construct histograms of translocation rates, except for MDA5 with ADPCP and the MDA5∆N–LGP2 complexes, due to their rates being too close to zero.

### Peak intensity analysis

To determine the peak intensity of the MDA5 on 1000 bp dsRNA substrate, single-molecule movies were recorded for 30 min. MDA5 molecules were tracked by SPARTAN to generate trajectories and peak intensities.

### Binning method

All binned histograms were produced by automatically splitting the data range into bins of equal size by using the Origin program.

### Molecular modeling of an MDA5–LGP2 complex

The MDA5–LGP2–dsRNA model was obtained using AlphaFold3 server.

## Supplementary information

Supplementary Information text summary

Supplementary information, Figure S1

Supplementary information, Figure S2

Supplementary information, Figure S3

Supplementary information, Figure S4

Supplementary information, Figure S5

Supplementary information, Figure S6

Supplementary information, Table S1

Supplementary information, Table S2

Supplementary information, Table S3

Supplementary information, Video S1

Supplementary information, Video S2

Text Note of Videos
