# Supplementary material for: ATP-dependent one-dimensional movement maintains immune homeostasis by suppressing spontaneous MDA5 filament assembly
Source: Cell Res. 2025 Sep 19;35(11):900–12. doi: 10.1038/s41422-025-01183-8 (PMC12589613; doi:10.1038/s41422-025-01183-8)
Supplement: Supplementary file 1 — Supplementary Information text summary [file 41422_2025_1183_MOESM1_ESM.pdf]

Supplementary Information includes:

Figures S1-S6 (PDF)
Tables S1-S3 (PDF)
Video S1 and S2 (MP4)
Text Notes of Videos (PDF)
