# Supplementary material for: ATP-dependent one-dimensional movement maintains immune homeostasis by suppressing spontaneous MDA5 filament assembly
Source: Cell Res. 2025 Sep 19;35(11):900–12. doi: 10.1038/s41422-025-01183-8 (PMC12589613; doi:10.1038/s41422-025-01183-8)
Supplement: Supplementary file 2 — Supplementary information, Figure S1 [file 41422_2025_1183_MOESM2_ESM.pdf]

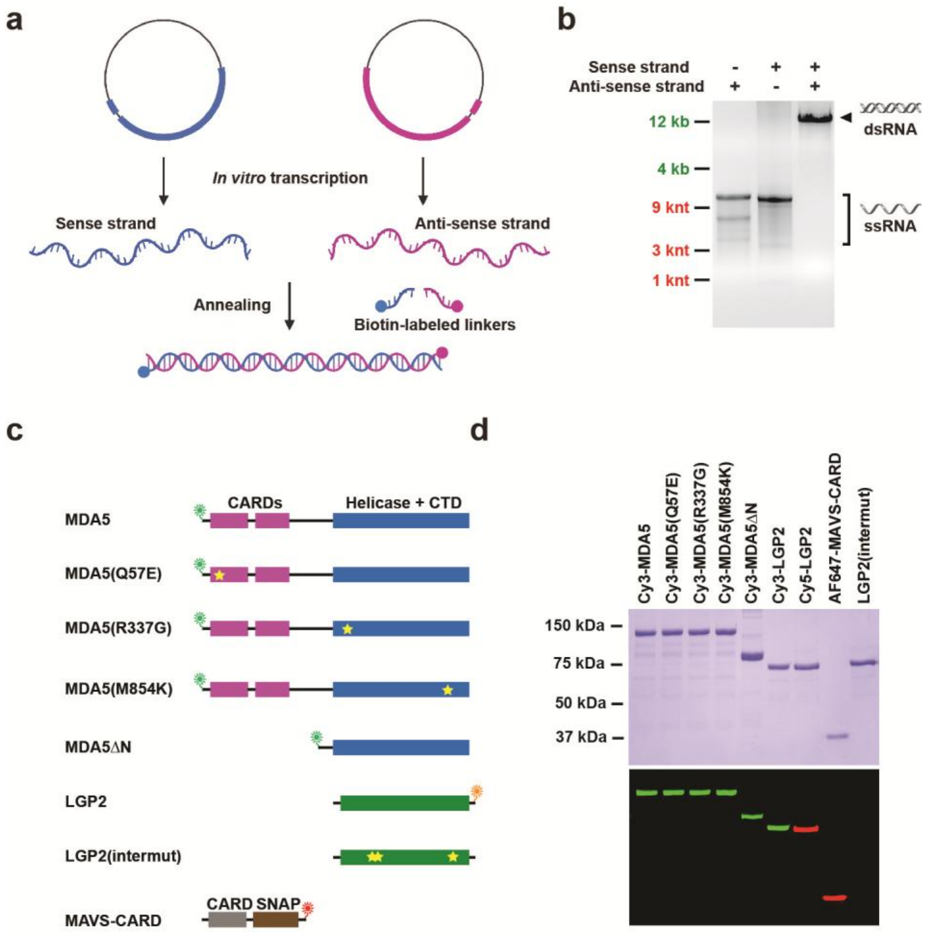


**Fig. S1 The construction of 11.6-kb dsRNA and fluorophore-labeled proteins used in smTIRF microscopy. a** A schematic illustration for the construction of an 11.6-kb dsRNA. Sense and anti-sense strands of ssRNA were annealed with two biotin-labeled RNA linkers to generate 11.6-kb dsRNA. **b** Agarose gel (0.75 %) showing the sense strand, anti-sense strand of ssRNA and the annealed 11.6-kb dsRNA. **c** A schematic illustration of labeled and unlabeled proteins used in single-molecule studies. **d** Coomassie stained (top) and fluorescent (bottom) images of SDS-PAGE gels showing the purified proteins.
