# Supplementary material for: ATP-dependent one-dimensional movement maintains immune homeostasis by suppressing spontaneous MDA5 filament assembly
Source: Cell Res. 2025 Sep 19;35(11):900–12. doi: 10.1038/s41422-025-01183-8 (PMC12589613; doi:10.1038/s41422-025-01183-8)
Supplement: Supplementary file 3 — Supplementary information, Figure S2 [file 41422_2025_1183_MOESM3_ESM.pdf]

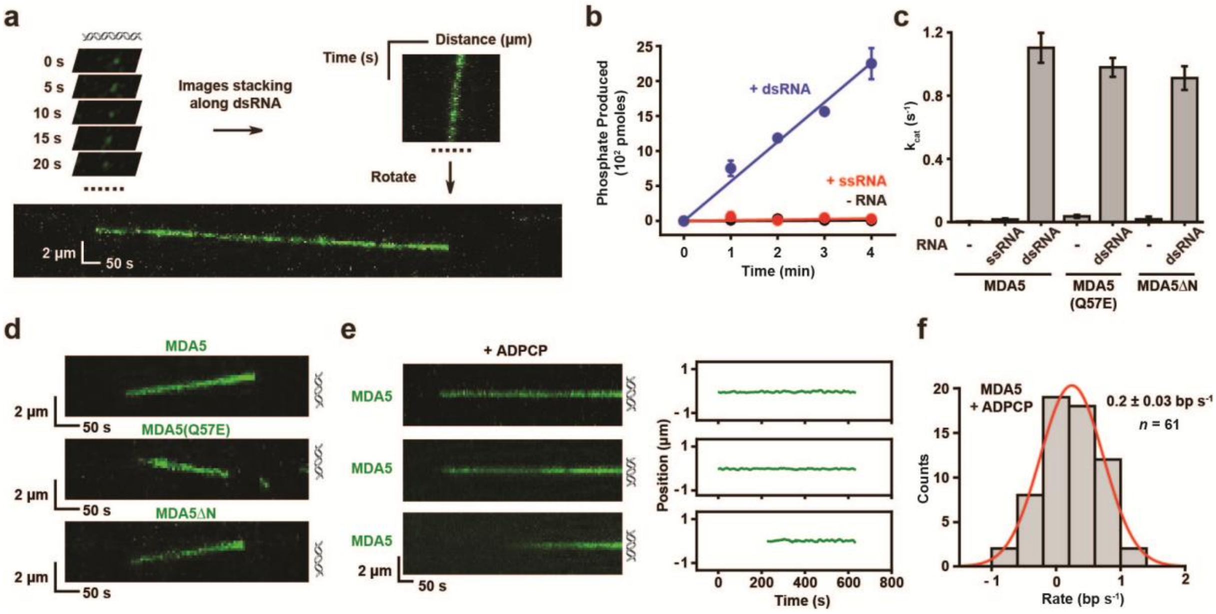

Fig. S2 ATPase activities and representative kymographs of MDA5 motors. a An illustration of the kymograph construction of a single MDA5 motor on 11.6-kb dsRNA. **b** ATP hydrolysis of MDA5 measured at various time using different RNA substrates. Circles represent individual numbers from at least three independent experiments (error bars: mean ± s.e.). A linear function was fit to the data to derive the turnover number (k_{cat}) of MDA5 ATPase (mean ± s.e.). **c** The turnover numbers (k_{cat}) of MDA5, MDA5(Q57E) and MDA5ΔN ATPase using different RNA substrates (error bars: mean ± s.e.). **d** Representative kymographs showing the translocation of MDA5, MDA5(Q57E) and MDA5ΔN (3 nM) on dsRNA. **e** Representative kymographs (left) and single-particle trajectories (right) showing the immobile binding of MDA5 (3 nM) on dsRNA with ADPCP. Positions of dsRNA are shown adjacent to the right of kymographs. **f** Histogram of binned MDA5 translocation rates with ADPCP. Data were fit to Gaussian function to derive the average rates (mean ± s.d.; *n* = number of events).
