# Supplementary material for: ATP-dependent one-dimensional movement maintains immune homeostasis by suppressing spontaneous MDA5 filament assembly
Source: Cell Res. 2025 Sep 19;35(11):900–12. doi: 10.1038/s41422-025-01183-8 (PMC12589613; doi:10.1038/s41422-025-01183-8)
Supplement: Supplementary file 4 — Supplementary information, Figure S3 [file 41422_2025_1183_MOESM4_ESM.pdf]

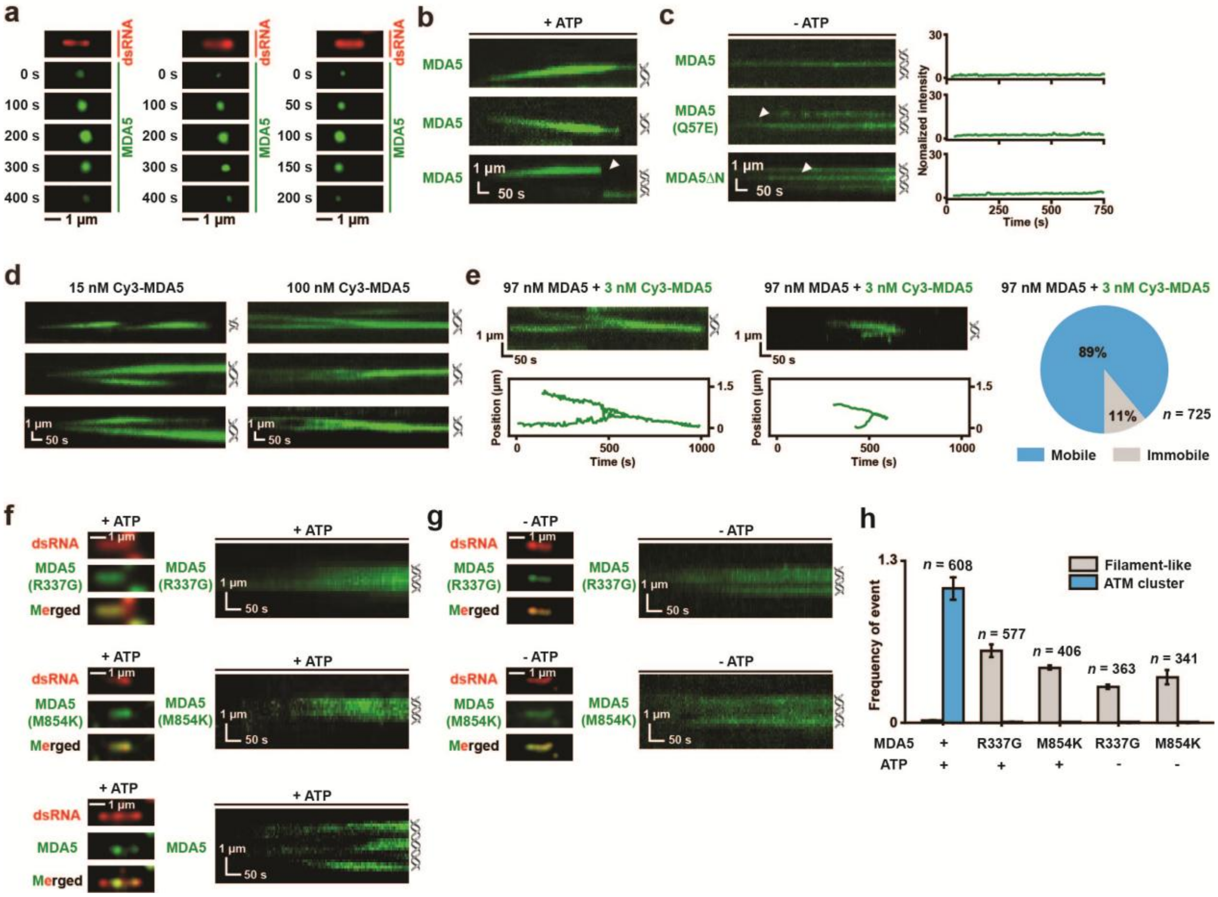

Fig. S3. Representative images, kymographs, trajectories and filament-like structure frequencies of MDA5(R337G) and MDA5(M854K). a Representative fluorescent images showing the formation of mobile MDA5 foci (15 nM). SYBR Gold stained 11.6-kb dsRNA is shown in red and Cy3-MDA5 at various times is shown in green. **b** Representative kymographs showing the formation of mobile MDA5 foci (15 nM). Arrowhead indicates the displacement of neutravidin from a dsRNA end. Positions of dsRNA are shown adjacent to the right of kymographs. **c** Representative kymographs (left) and time-dependent fluorescent intensities (right) showing the absence of MDA5 mobile foci without ATP. Cy3-MDA5 (15 nM) is shown in green. Arrowheads indicate the MDA5 molecules used for fluorescent intensity plots. Positions of dsRNA are shown adjacent to the right of kymographs. **d** Representative kymographs showing the presence/absence of MDA5 collision event at various concentrations. **e** Left and middle: Representative kymographs and single-particle trajectories showing the behavior of individual motors within colliding foci. Right: Pie charts showing the distributions of mobile and immobile MDA5 at 100 nM protein concentration (*n* = number of MDA5 molecules). **f** Representative fluorescent images (left) and kymographs (right) showing the binding of MDA5, MDA5(R337G) and MDA5(M854K) (100 nM) on dsRNA with ATP. SYBR Gold stained 11.6-kb dsRNA is shown in red and Cy3-MDA5 is shown in green. Positions of dsRNA are shown adjacent to the right of kymograph. **g** Representative fluorescent images (left) and kymographs (right) showing the binding of MDA5(R337G) and MDA5(M854K) (100 nM) on dsRNA without ATP. SYBR Gold stained 11.6-kb dsRNA is shown in red and Cy3-MDA5 is shown in green. Positions of dsRNA are shown adjacent to the right of kymograph. **h** Frequency of filament-like structure and ATM cluster of MDA5, MDA5(R337G) and MDA5(M854K) (mean ± s.d.; *n* = number of dsRNA molecules).
