# Supplementary material for: ATP-dependent one-dimensional movement maintains immune homeostasis by suppressing spontaneous MDA5 filament assembly
Source: Cell Res. 2025 Sep 19;35(11):900–12. doi: 10.1038/s41422-025-01183-8 (PMC12589613; doi:10.1038/s41422-025-01183-8)
Supplement: Supplementary file 5 — Supplementary information, Figure S4 [file 41422_2025_1183_MOESM5_ESM.pdf]

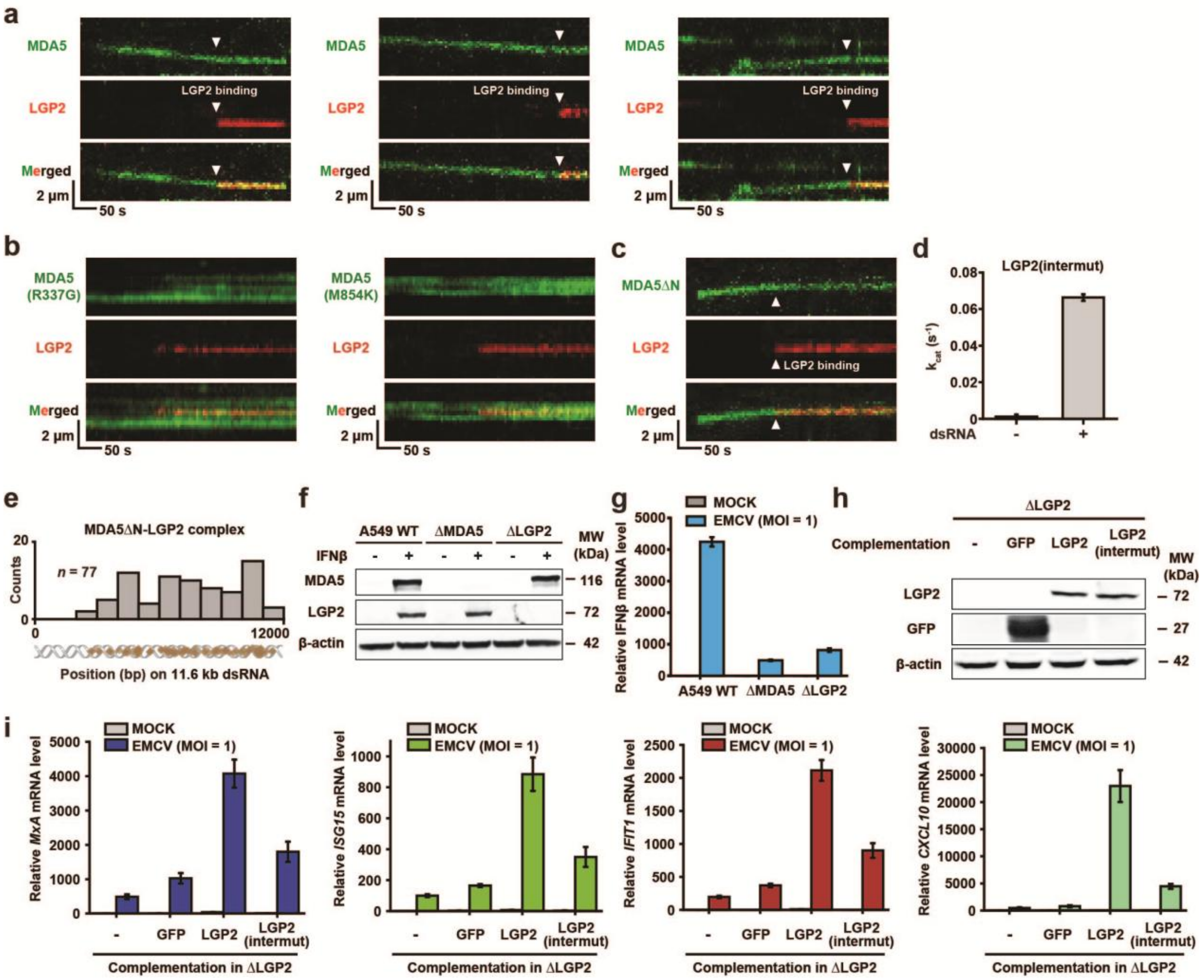

Fig. S4. Representative kymographs, ATPase activity, immunoblotting gels and MDA5 signaling. a Representative kymographs showing Cy5-LGP2 (3 nM) binding to Cy3-MDA5 motors (3 nM). Cy3-MDA5 are shown in green and Cy5-LGP2 are shown in red. Merged kymographs are generated by overlaying both channels. Arrowheads indicate the association of LGP2 with MDA5. **b** Representative kymographs showing Cy5-LGP2 (30 nM) binding to Cy3-MDA5(R337G) or Cy3-MDA5(M854K) (30 nM). **c** Representative kymographs showing a Cy5-LGP2 (0.5 nM) binding to a Cy3-MDA5ΔN motor (0.5 nM). Arrowheads indicate the association of LGP2 with MDA5ΔN. **d** The turnover numbers (k_{cat}) of LGP2(intermut) ATPase using 11.6-kb dsRNA substrate (error bars: mean ± s.e.). **e** Distribution of the positions for MDA5ΔN-LGP2 complex formation on dsRNA (n = number of events). Diamonds represent individual events. **f** Immunoblotting showing the successful knockouts of MDA5/LGP2 in A549 cells. Proteins were detected using indicated antibodies. Endogenous MDA5/LGP2 expression was induced by interferon stimulation. **g** Relative IFNβ mRNA levels showing the MDA5 signaling activities in *wild-type* A549, ΔMDA5 and ΔLGP2 cells after 24 h EMCV infection. MOI: multiplicity of infection. **h** Immunoblotting showing the overexpression of GFP, LGP2 and LGP2(intermut) in A549ΔLGP2 cells. **i** Relative MxA, ISG15, IFIT1 and CXCL10 mRNA levels showing the MDA5 signaling activity in ΔLGP2 cells. MOI: multiplicity of infection.
