# Supplementary material for: ATP-dependent one-dimensional movement maintains immune homeostasis by suppressing spontaneous MDA5 filament assembly
Source: Cell Res. 2025 Sep 19;35(11):900–12. doi: 10.1038/s41422-025-01183-8 (PMC12589613; doi:10.1038/s41422-025-01183-8)
Supplement: Supplementary file 6 — Supplementary information, Figure S5 [file 41422_2025_1183_MOESM6_ESM.pdf]

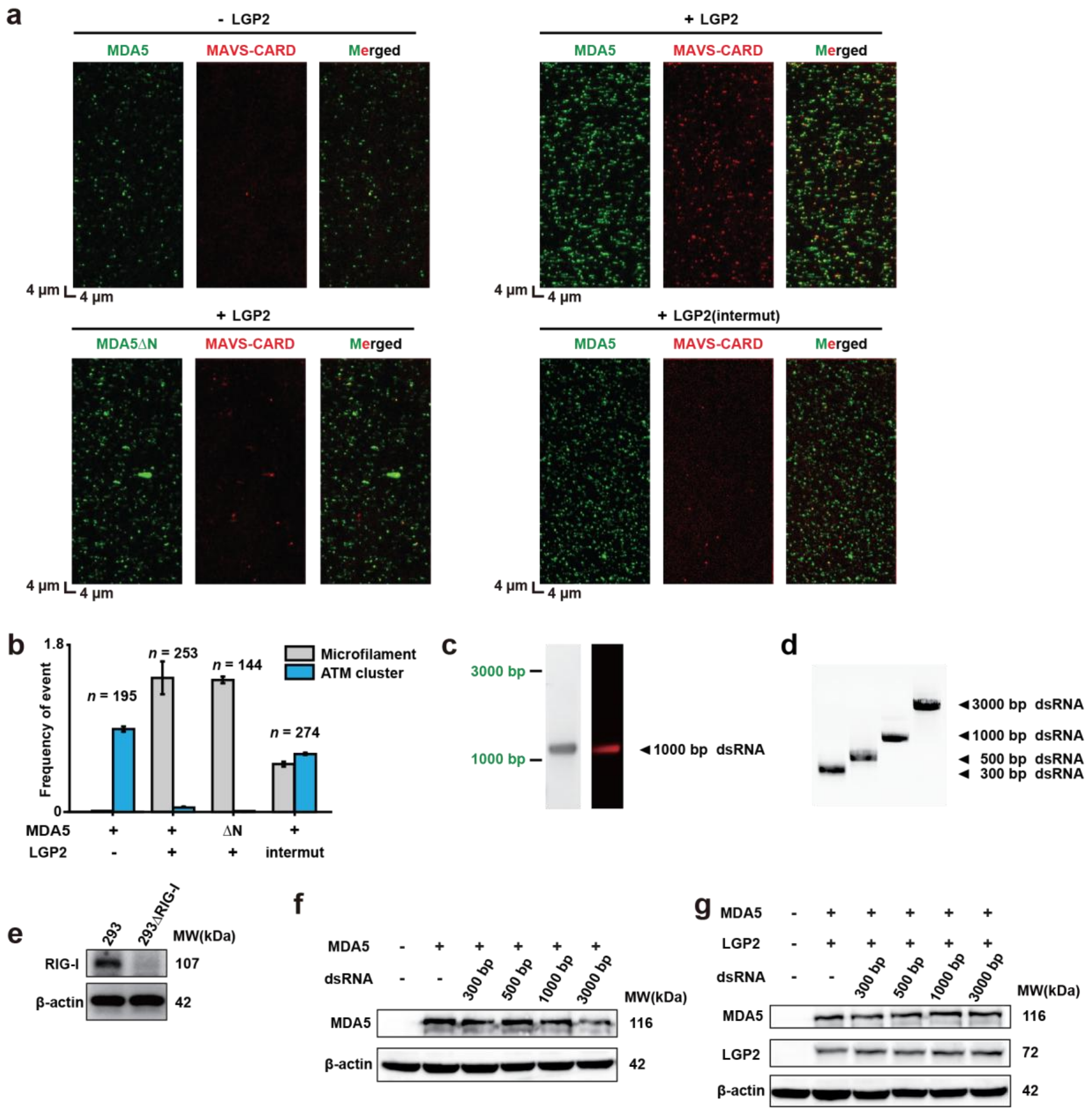

Fig. S5 Images of MAVS-CARD recruitments by MDA5, RNA and immunoblotting gels. a Representative images showing the recruitments of AF647-MAVS-CARD (100 nM) by Cy3-MDA5 or Cy3-MDA5ΔN (20 nM) under various conditions. The absence or presence of LGP2 (20 nM) is indicated above each set of images. **b** The frequency of immobile and mobile MDA5 under various conditions (mean ± s.d.; *n* = number of dsRNA molecules). **c** Ethidium bromide stained (left) and fluorescent (right) agarose gel showing Cy5-labeled 1000 bp dsRNA substrates. **d** Ethidium bromide stained agarose gel showing 300 bp, 500 bp, 1000 bp and 3000 bp dsRNA substrates. **e** Immunoblotting showing the successful knockout of RIG-I in 293ΔRIG-I. **f** Immunoblotting showing the overexpression of MDA5 in 293ΔRIG-I. **g** Immunoblotting showing the simultaneous overexpression of MDA5 and LGP2 in 293ΔRIG-I.
