# Supplementary material for: ATP-dependent one-dimensional movement maintains immune homeostasis by suppressing spontaneous MDA5 filament assembly
Source: Cell Res. 2025 Sep 19;35(11):900–12. doi: 10.1038/s41422-025-01183-8 (PMC12589613; doi:10.1038/s41422-025-01183-8)
Supplement: Supplementary file 7 — Supplementary information, Figure S6 [file 41422_2025_1183_MOESM7_ESM.pdf]

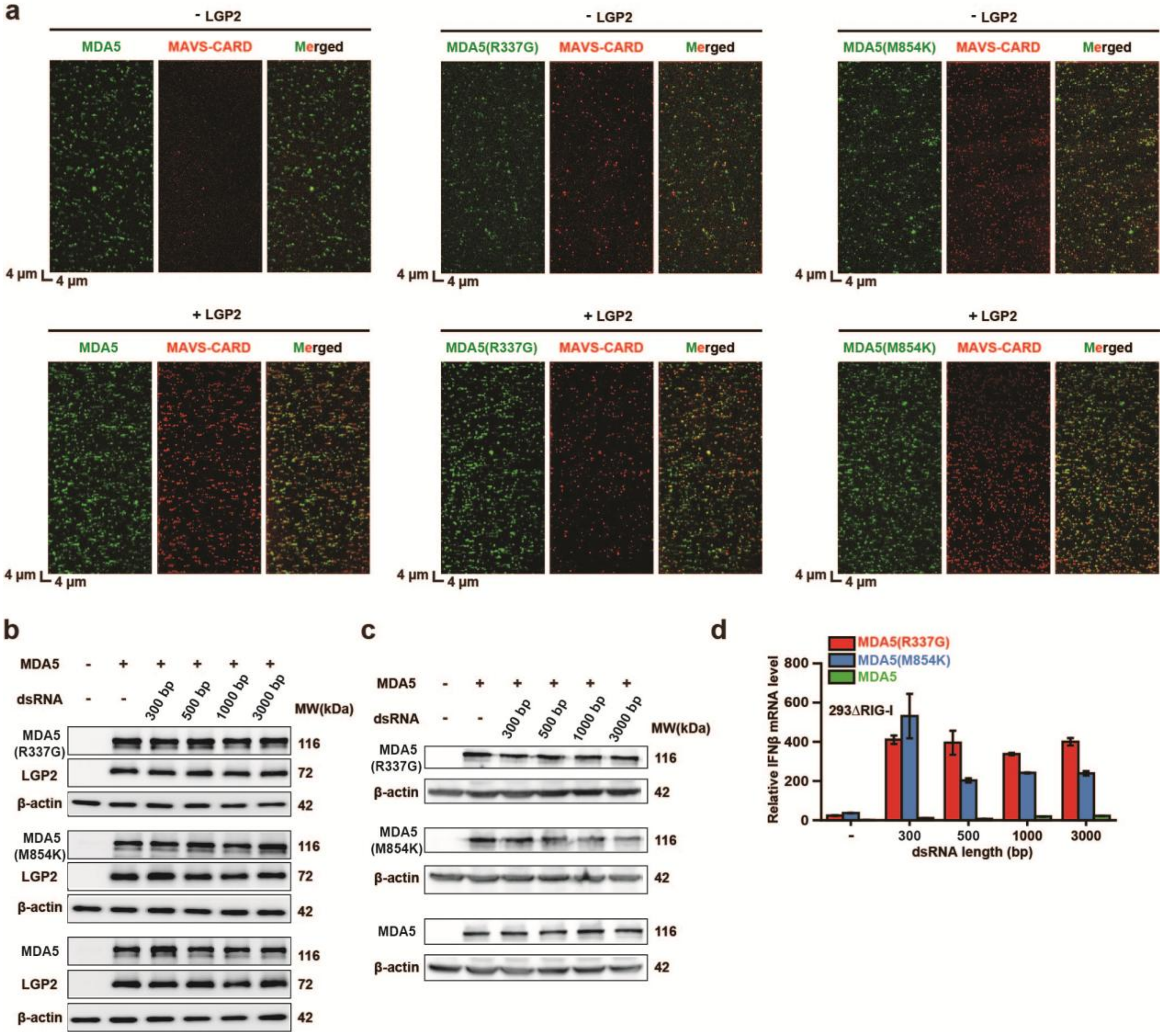

Fig. S6. Images of MAVS-CARD recruitments by MDA5, immunoblotting gels and representative kymographs of mMDA5. **a** Representative images showing the recruitments of AF647-MAVS-CARD (100 nM) by Cy3-MDA5, Cy3-MDA5(R337G) or Cy3-MDA5(M854K) proteins (100 nM). The absence or presence of LGP2 (100 nM) is indicated above each set of images. **b** Immunoblotting showing the co-expression of MDA5(R337G), MDA5(M854K) and MDA5 proteins with LGP2 in 293ΔRIG-I. **c** Immunoblotting showing the overexpression of MDA5(R337G), MDA5(M854K) and MDA5 proteins in 293ΔRIG-I. **d** Column plots of relative IFNβ mRNA levels showing the MDA5 signaling activity under various conditions. 293ΔRIG-I with MDA5(R337G) overexpression [MDA5(R337G)], 293ΔRIG-I with MDA5(M854K) overexpression [MDA5(M854K)] and 293ΔRIG-I with MDA5 overexpression (MDA5) were stimulated with dsRNA substrates of different lengths.
