# Supplementary material for: ATP-dependent one-dimensional movement maintains immune homeostasis by suppressing spontaneous MDA5 filament assembly
Source: Cell Res. 2025 Sep 19;35(11):900–12. doi: 10.1038/s41422-025-01183-8 (PMC12589613; doi:10.1038/s41422-025-01183-8)
Supplement: Supplementary file 8 — Supplementary information, Table S1 [file 41422_2025_1183_MOESM8_ESM.pdf]

Table S1. Oligonucleotides used in this study

Name	Sequences (5'-3')
MDA5 for1	ATCACAGCAGCCTCCCTGAAACTGGAGGAGGGTCCATGTCGAATGGGTAT
MDA5 for2	CAGAGAGGATCCATGCATCATCATCATCATCACAGCAGCCTC
MDA5 rev	AAGCTTGGTACCTAATCCTCATCACTAAATAAAC
MDA5(Q57E) for	GAGGCAGTTGAACTGCTGCTGAGCA
MDA5(Q57E) rev	CATGTTCCCGGAGGTGGCGACTGTC
MDA5(R337G) for	GGAGTGGCTGTTTACATTGCCAAGG
MDA5(R337G) rev	GGTTTTTCCACTCCCTGTAGGGAGG
MDA5(M854K) for	AAATATAAAGCTATACATTGTGTTC
MDA5(M854K) rev	CATCTTCTCTCGGAAATCATTAACT
MDA5ΔN for	ATGGGAAGTGATTCAGATGAAGAGA
MDA5ΔN rev	GGACCCTCCTCCAGTTTCAGGGAG
LGP2 for	CCCACCATCGGGCGCGGATCCATGGAGCTTCGGTCATACC
LGP2 rev1	GGCTGCTACCGGTTTCCGGCAGGGATCCACCACCACCACCACCACCGTCCAGGGAGAGGT
LGP2 rev2	CCTTTGAATTCCGCGCGCTTCGGACCGTTAATGATGATGATGATGATGGCTGCTACCGGTT
LGP2(intermut) for1	GCAGCAGAGCACGTGGAGCTCACTGTC
LGP2(intermut) for2	GCATGGAAGCCTGGGGGTGTCATC
LGP2(intermut) rev1	CTCGGGGCTGGTCAGTGCCATCTG
LGP2(intermut) rev2	CTTGAAGACTTTGTTGATGACCAC
MAVS-CARD for1	AAGGTTGTAAAGATTCTGCCTTACCTGCCCTGCCT
MAVS-CARD for2	AAGCTAGTTGATCTCGCGGACGAAG
MAVS-CARD rev1	CACATTGCAAAAATTGCTGAAATTG
MAVS-CARD rev2	ACAGCCCCTCAGTGCCGCAATGAAG
RNA linker 1	CCGGAUCGCUCGAGACGCAUUUGCAUCUAGAGGGCCCUAUUC-biotin
RNA linker 2	GUACCGCGCUGAUAAGCCUGGUUGACGGAAGUGGCAAUUCUAGAGGGCCCUAUUC-biotin
RNA linker 3	Cy5-UGAGGAUCCCGGAUCGCUCGAGACGCAUUUGCAUCUAGAGGGCCCUAUUC-biotin
ssRNA31	UUUUUUGGGUUUUCCCAGUCACGACGUUGUA

Table S1. Oligonucleotides used in this study (continued)

Name	Sequences (5'-3')
qPCR *IFNB* for	CTTTCGAAGCCTTTGCTCTG
qPCR *IFNB* rev	CAGGAGAGCAATTTGGAGGA
qPCR *ACTB* for	CACTCTTCCAGCCTTCCTTC
qPCR *ACTB* rev	TACAGGTCTTTGCGGATGTC
qPCR *IFIT1* for	TTGATGACGATGAAATGCCTGA
qPCR *IFIT1* rev	CAGGTCACCAGACTCCTCAC
qPCR *ISG15* for	CTCTGAGCATCCTGGTGAGGAA
qPCR *ISG15* rev	AAGGTCAGCCAGAACAGGTCGT
qPCR *MxA* for	ACAGGACCATCGGAATCTTG
qPCR *MxA* rev	CCCTTCTTCAGGTGGAACAC
qPCR *CXCL10* for	GTGGCATTCAAGGAGTACCTC
qPCR *CXCL10* rev	TGATGGCCTTCGATTCTGGATT
qPCR m*Ifnb* for	CCCTATGGAGATGACGGAGA
qPCR m*Ifnb* rev	CTGTCTGCTGGTGGAGTTCA
qPCR m*Actb* for	TGACGTTGACATCCGTAAAGACC
qPCR m*Actb* rev	AAGGGTGTAAAACGCAGCTCA
qPCR m*Ifit1* for	CTGAGATGTCACTTCACATGGAA
qPCR m*Ifit1* rev	GTGCATCCCCAATGGGTTCT
qPCR m*Cxcl10* for	GGTCTGAGTGGGACTCAAGG
qPCR m*Cxcl10* rev	GTGGCAATGATCTCAACACG
