# Supplementary material for: ATP-dependent one-dimensional movement maintains immune homeostasis by suppressing spontaneous MDA5 filament assembly
Source: Cell Res. 2025 Sep 19;35(11):900–12. doi: 10.1038/s41422-025-01183-8 (PMC12589613; doi:10.1038/s41422-025-01183-8)
Supplement: Supplementary file 9 — Supplementary information, Table S2 [file 41422_2025_1183_MOESM9_ESM.pdf]

Table S2. Labeling efficiencies of proteins

Protein	Labeling Efficiencies
Cy3 labeled MDA5	50%
Cy3 labeled MDA5(Q57E)	49%
Cy3 labeled MDA5ΔN	30%
Cy3 labeled MDA5(R337G)	45%
Cy3 labeled MDA5(M854K)	45%
Cy3 labeled LGP2	55%
Cy5 labeled LGP2	39%
AF647 labeled MAVS-CARD	49%
