# Supplementary material for: ATP-dependent one-dimensional movement maintains immune homeostasis by suppressing spontaneous MDA5 filament assembly
Source: Cell Res. 2025 Sep 19;35(11):900–12. doi: 10.1038/s41422-025-01183-8 (PMC12589613; doi:10.1038/s41422-025-01183-8)
Supplement: Supplementary file 10 — Supplementary information, Table S3 [file 41422_2025_1183_MOESM10_ESM.pdf]

Table S3. The frequency of MDA5 varieties observed by single-molecule analysis

Protein	ATP	Frequency of static binding	Frequency of translocation
MDA5	-	0.136±0.0554	0
	+	0.022±0.0039	0.114±0.0085
	ADPCP	0.337±0.0424	0
MDA5(Q57E)	+	0.086±0.0162	0.527±0.0064
MDA5ΔN	+	0.105±0.0279	1.420±0.0849
