# Supplementary material for: ATP-dependent one-dimensional movement maintains immune homeostasis by suppressing spontaneous MDA5 filament assembly
Source: Cell Res. 2025 Sep 19;35(11):900–12. doi: 10.1038/s41422-025-01183-8 (PMC12589613; doi:10.1038/s41422-025-01183-8)
Supplement: Supplementary file 13 — Text Note of Videos [file 41422_2025_1183_MOESM13_ESM.pdf]

Text Note of Video S1
Representative movie (20 frames per sec) of an MDA5 motor translocating on dsRNA (Fig. 1c, middle). Two channels were merged. MDA5 is shown as green and dsRNA is shown as red.

Text Note of Video S2
Representative movie (20 frames per sec) of an MDA5 ATM cluster formation on dsRNA. Two channels were merged. MDA5 is shown as green and dsRNA is shown as red.
